# Nurse-Led and Nurse-Involved Antimicrobial Stewardship in Hospitals and Primary Care: Protocol for a Mixed Methods Systematic Review and Meta-Analysis With Gulf Cooperation Council and Middle East and North Africa Subgroup Analysis

**DOI:** 10.2196/96342

**Published:** 2026-10-08

**Authors:** Suthan Pandarakutty, Brenda Poku, Shanthi Ramasubramaniam, Sylivia Nalubega, Mansour Mansour, Manju Varghese, Lakshmi Renganathan, Vijith Chandu, Virgina Varghese, Faiza Al Zadjali, Vishnu Renjith

**Affiliations:** 1 Department of Nursing, Fatima College of Health Sciences, Abu Dhabi, United Arab Emirates; 2 School of Sociology and Social Policy, University of Nottingham, Law and Social Science Building, University Park, Nottingham, England, NG3 3EW, United Kingdom, 44 7974103283; 3 School of Health and Social Well-Being, Faculty of Health and Applied Sciences, University of the West of England, Bristol, England, United Kingdom; 4 Ministry of Health, Oman College of Health Sciences, Muscat, Oman; 5 College of Applied and Health Sciences, A’Sharqiyah University, Ibra, Oman; 6 Oman Medical Specialty Board, Muscat, Oman; 7 School of Nursing and Midwifery, Royal College of Surgeons in Ireland, Dublin, Leinster, Ireland

**Keywords:** antimicrobial stewardship, nurse-led, mixed methods systematic review, meta-analysis, GRADE, implementation science, protocol, Gulf Cooperation Council, barriers and facilitators, antibiotic consumption

## Abstract

**Background:**

Antimicrobial resistance poses a growing threat to patient safety worldwide. Nurses are central to antimicrobial management; however, their contribution to formal antimicrobial stewardship (AMS) programs remains poorly characterized. To our knowledge, no previous review has combined evidence on the effectiveness and implementation of nurse-led and nurse-involved AMS interventions, including Gulf Cooperation Council and Middle East and North Africa (GCC/MENA) subgroup analysis.

**Objective:**

This protocol describes a mixed methods systematic review that aims to determine (1) the effectiveness of nurse-led or nurse-involved AMS interventions on patient and antimicrobial outcomes in hospital and primary care settings and (2) the barriers and facilitators influencing this implementation using the Consolidated Framework for Implementation Research (CFIR 2.0).

**Methods:**

This review follows the Joanna Briggs Institute convergent, parallel-streams mixed methods methodology. Eligible studies report a nurse-led or nurse-involved AMS intervention among adults receiving antimicrobial therapy in hospital or primary care settings; pediatric-only and long-term care settings are excluded. Eleven databases will be searched from January 2000 to the calendar month of searching. Two reviewers will independently screen records in 2 rounds, with agreement quantified by Cohen κ (target ≥0.70). The quantitative stream will include randomized, quasi-experimental, interrupted time series, and controlled before-and-after studies, with effect measures matched to outcome type. Risk of bias will be independently assessed using design-matched tools. Random-effects meta-analysis will be the primary approach, with prediction intervals supplementing *I*² to quantify heterogeneity. The qualitative stream will synthesize implementation data through hybrid deductive-inductive CFIR 2.0 thematic synthesis. Both streams will be integrated through a convergent joint display classifying findings as convergent, complementary, expansive, or discordant. Three primary subgroup analyses will compare GCC/MENA vs the rest of the world, hospital vs primary care, and levels of nursing involvement. GRADE (Grading of Recommendations Assessment, Development and Evaluation) will be applied to all primary quantitative outcomes.

**Results:**

Database searches, Covidence import, and deduplication were completed in March 2026. Title and abstract screening, including pilot calibration, was completed by June 2026. Full-text screening was planned for completion by August 2026, followed by parallel quantitative and qualitative data extraction from August to September 2026. Meta-analysis, CFIR thematic synthesis, and GRADE assessment are expected by November 2026, with mixed methods integration planned for November to December 2026. Manuscript writing is planned for January to February 2027, with submission targeted for March 2027. No amendments to the registered protocol have occurred.

**Conclusions:**

This review is intended to provide one of the first meta-analytic syntheses of nurse-led and nurse-involved AMS clinical effectiveness alongside a structured implementation analysis. Findings may inform stewardship program design, nursing education, and health policy, particularly in GCC/MENA settings where nursing AMS evidence is scarce.

## Introduction

### Background

Antimicrobial resistance (AMR) has emerged as a significant and worsening threat to global public health, threatening the effectiveness of modern medicine and the sustainable prevention and treatment of infectious diseases [1]. A landmark analysis from the 2019 Global Burden of Disease study placed the toll of resistant infections at nearly 5 million deaths in a single year, with over 1.27 million of those deaths directly attributable to resistance rather than the underlying infection [2]. Projections from the O’Neill review [3] estimate that, without coordinated global action, drug-resistant infections could claim 10 million lives annually by 2050.

A key part of the global AMR response has been the growth of antimicrobial stewardship (AMS) programs (ASPs), which are coordinated efforts to ensure that antibiotics are used at the right dose, via the right route, and for the right duration, with the broader aim of reducing inappropriate prescribing and slowing the development of resistance [4]. Published guidelines from the Infectious Diseases Society of America and the Society for Healthcare Epidemiology of America set a practical framework for institutional ASP implementation in 2007, with a revised version in 2016 establishing the current standard [5]. Evidence suggests that well-implemented ASPs can reduce overall and inappropriate antimicrobial use, lower antimicrobial-related costs, decrease *Clostridioides difficile*–associated disease, and, in some studies, reduce the emergence of AMR [6]. Despite this evidence base, ASPs in most settings remain predominantly physician-led and pharmacist-led, with nursing contributions acknowledged informally at best.

Nurses represent the largest professional group in the global health workforce, numbering approximately 29.8 million worldwide in 2023, an increase from 27.9 million in 2018 [7]. Nurses contribute to medication administration, patient monitoring, patient and family education, and communication across the health care team [8]. These roles place nurses at the center of day-to-day antimicrobial care and point to a largely underused contribution to formal stewardship programs.

Evidence confirms that nursing contributions to AMS often occur informally and remain poorly recognized. An integrative review by Monsees et al [9] identified nursing knowledge, education, and information needs, as well as patient safety and organizational factors influencing antibiotic management. Qualitative studies indicate that nurses are well positioned to contribute to AMS through their patient advocacy and infection management roles; however, their participation may be limited by traditional perceptions of nursing responsibilities, interprofessional tensions, insufficient education, limited integration into AMS activities, and organizational or resource constraints [10,11].

Textbox 1 lists the AMS-relevant activities that inform the nursing-involvement threshold used throughout this protocol. To align the box explicitly with the eligibility criteria and search strategy described in the *Methods* section, an included study must attribute at least one of these activities explicitly to a nurse, either as an independent action or as a designated, protocol-driven component of a multidisciplinary intervention.

These activities constitute the minimum nursing involvement threshold for inclusion in this review’s quantitative stream (stream 1). Qualitative studies documenting any of these contributions even where formal outcome data are absent are eligible for the implementation synthesis stream (stream 2).

Textbox 1. **Nurse-led antimicrobial stewardship (AMS) activities (included interventions).**

- Antibiotic allergy verification and delabeling support: reviewing documented antibiotic allergy histories, particularly penicillin allergy labels, and referring patients for appropriate assessment to reduce unnecessary use of broad-spectrum or alternative antibiotics [12]
- Intravenous-to-oral switch support: identifying clinically stable patients who may be eligible to transition from intravenous to oral antimicrobial therapy and flagging them for prescriber review [13]
- Antimicrobial prescribing audit and feedback support: reviewing antimicrobial prescribing against relevant guidelines and contributing prescribing information to structured audit-and-feedback processes within the multidisciplinary stewardship team [14,15]
- Blood culture collection before the first antibiotic dose: ensuring microbiological specimens are obtained before antimicrobial therapy begins, enabling culture-guided treatment [15]
- Monitoring for signs of treatment failure: recognizing clinical deterioration, inadequate microbiological response, or antibiotic-related adverse effects and escalating appropriately [9]
- Monitoring for antibiotic-related adverse events: observing patients for gastrointestinal, renal, hematologic, dermatologic, neurologic, and other adverse effects associated with antibiotic therapy and escalating concerns appropriately [16]
- Antimicrobial prescribing audit and feedback support: reviewing antimicrobial prescribing against relevant guidelines and contributing prescribing information to structured audit-and-feedback processes within the multidisciplinary stewardship team [15]
- De-escalation support: monitoring culture and sensitivity results and proactively identifying opportunities to switch from broad-spectrum to narrower-spectrum agents once a causative organism is confirmed [14]
- Surgical antimicrobial prophylaxis support: contributing to standardized surgical antimicrobial prophylaxis workflows, documentation, review, and follow-up within the multidisciplinary team [17]
- Sepsis bundle management with AMS component: initiating nurse-driven rapid sepsis responses that include timely antibiotic administration and culture collection before the first dose [15]

Where formal nursing roles in AMS have been studied, the evidence of clinical benefit is encouraging. Structured antibiotic timeout protocols, intravenous-to-oral switch screening, nurse-led patient education, and nurse-initiated blood culture collection have each been associated with reductions in antibiotic exposure and improvements in prescribing appropriateness [17,18]. A multisite survey found that nurses’ contribution to AMS is frequently underused relative to their opportunity to contribute [19]. However, these positive signals exist within an evidence base that is fragmented, methodologically inconsistent, and has never been synthesized through meta-analysis.

A recent mapping review applying the Nursing Role Effectiveness Model (NREM) framework found that only 9% of included studies on nursing and AMS evaluated the impact of nurse-led practices on actual clinical or antimicrobial outcomes [20]. The complementary PROSPERO (International Prospective Register of Systematic Reviews)-registered systematic reviews on nurses’ knowledge and perceptions [21] and nurses’ qualitative experiences of AMS [22] similarly confirm that clinical effectiveness has not been systematically answered.

This gap is particularly significant for the Gulf Cooperation Council and Middle East and North Africa (GCC/MENA) regions. For the purposes of this review, GCC/MENA refers to the 6 Gulf Cooperation Council member states (Bahrain, Kuwait, Oman, Qatar, Saudi Arabia, and the United Arab Emirates) together with the World Bank’s Middle East and North Africa classification (Algeria, Djibouti, Egypt, Iran, Iraq, Israel, Jordan, Lebanon, Libya, Morocco, Syria, Tunisia, West Bank and Gaza, and Yemen) [23]. High rates of multidrug-resistant Gram-negative organism carriage have been reported across the Arabian Peninsula [24]. Systematic reviews of ASP implementation in Middle Eastern countries [25] and GCC states [26] have consistently noted that nursing contributions are absent from, or marginalized within, the regional AMS literature. No existing review has conducted a prespecified subgroup analysis for GCC/MENA nurse-led AMS activity.

Relatively little is also known about why nurse-led AMS interventions succeed in some settings but not others. The Consolidated Framework for Implementation Research (CFIR 2.0), comprising 5 implementation domains (described in Textbox 2 under the *Methods* section) [27], has been applied in recent AMS implementation research [28,29] and, most recently, in other health care implementation contexts, such as digital service adoption among nurses and physicians [30], but not yet used to systematically explain variation in nurse-led AMS implementation across health care systems or regions.

This review draws together available evidence on the effectiveness and implementation of nurse-led and nurse-involved AMS interventions, with a dedicated subgroup analysis for the GCC/MENA region. To our knowledge, it will provide one of the first meta-analytic syntheses of clinical effectiveness combined with a CFIR 2.0-informed implementation analysis and a regional evidence estimate for GCC/MENA settings. Pediatric-only settings and nursing home or long-term care facilities are excluded from this review; while AMS is also relevant in these settings, nurse staffing models, prescribing pathways, and outcome measures differ sufficiently from adult acute and primary care settings that combining them would risk conflating distinct implementation contexts. This exclusion is discussed further under *Methods* and *Discussion* sections.

Textbox 2. **The Consolidated Framework for Implementation Research (CFIR 2.0): 5 domains [27].**

- Domain 1–innovation characteristics: Attributes of the antimicrobial stewardship (AMS) intervention itself that influence adoption: perceived relative advantage, complexity, adaptability to local context, and evidence-base credibility.
- Domain 2–outer setting: external contextual factors: national antimicrobial resistance (AMR) policies, regulatory or accreditation requirements, interorganizational pressures, and the broader epidemiology of AMR.
- Domain 3–inner setting: organizational characteristics: institutional culture, leadership support for nursing roles in AMS, staffing and resource availability, communication structures, and professional hierarchies.
- Domain 4–individuals: characteristics of those delivering and engaging with the intervention: nurses’ knowledge, beliefs about AMS, perceived professional responsibilities, and readiness to engage in formal stewardship activities.
- Domain 5–implementation process: activities to introduce and sustain the intervention: planning, engagement of nursing champions, training and capacity-building, monitoring and feedback, and long-term sustainability strategies.

### Research Questions

#### Primary Research Questions

This review will address the following primary research questions:

1. What is the effectiveness of nurse-led or nurse-involved AMS interventions on patient and antimicrobial outcomes in hospital and primary care settings globally? (quantitative stream)
2. What are the barriers and facilitators to implementing nurse-led or nurse-involved AMS interventions in hospital and primary care settings, as reported by health care professionals? (qualitative stream)

#### Secondary Research Questions

##### Stream 1 (Quantitative, Addressed by Meta-Analysis or Narrative Synthesis)

The following secondary research questions will be addressed in stream 1:

1. What nurse-led AMS tasks are described and operationalized in the published literature?
2. What is the effectiveness of nurse-led AMS tasks on antibiotic consumption, prescribing appropriateness, and clinical patient outcomes?
3. How does effectiveness differ by care setting (hospital inpatient vs primary care; intensive care unit [ICU] vs general ward)?
4. Among studies conducted in GCC/MENA countries, what nurse-led or nurse-involved AMS interventions have been reported, and what outcomes were measured?

##### Stream 2 (Qualitative, Addressed by CFIR 2.0 Thematic Synthesis)

The following secondary research questions will be addressed in stream 2:

1. Which CFIR 2.0 domains are most frequently reported as barriers or facilitators to implementing nurse-led AMS programs?
2. What are the perspectives of nurses, physicians, pharmacists, and managers regarding the nurse’s role in AMS and the factors that support or hinder intervention implementation?

## Methods

This protocol has been developed in accordance with the PRISMA-P (Preferred Reporting Items for Systematic Reviews and Meta-Analyses—Protocols) 2015 guidelines (Checklist 1) [31] and is registered on PROSPERO (CRD420261341653).

### Ethical Considerations

This is a protocol for a systematic review of published literature. No primary data collection involving human participants is planned.

### Study Design

We will use a Joanna Briggs Institute (JBI) convergent, parallel-streams, mixed methods systematic review design [32]. Quantitative and qualitative studies are searched, appraised, and analyzed separately in parallel streams before their outputs are integrated in a final convergent stage. This approach suits review questions requiring both an effectiveness answer (does nurse-led AMS work?) and an implementation answer (why does it work and under what conditions?). The 3-stage synthesis, stage A (quantitative meta-analysis), stage B (CFIR 2.0 qualitative synthesis), and stage C (joint display convergent integration) is described fully under the *Data Synthesis* section below.

### Nurse-Led and Nurse-Involved: A Prespecified Typology

Because “nurse-led” and “nurse-involved” interventions differ substantially in the degree of nursing autonomy they represent, this review applies a four-category typology to classify every eligible intervention at data extraction: (1) nurse-led, in which a nurse independently initiates, executes, and is accountable for the AMS task without requiring prescriber authorization for each instance; (2) nurse-initiated or semiautonomous, in which a nurse identifies and flags an AMS action but a prescriber retains sign-off authority; (3) nurse-involved with a defined AMS task, in which a nurse performs a specific, named AMS activity as a designated component of a multidisciplinary program, without independent decision-making authority; and (4) nurse-executed routine care without a formal AMS component, which does not meet the eligibility threshold and is excluded. This typology is aligned with the eligibility criteria below and will be reported descriptively for all included studies.

### Eligibility Criteria (PICOS Framework)

#### Population (P)

This review distinguishes 3 elements that are often conflated in the AMS literature: the clinical population (patients receiving antimicrobial therapy), the unit of analysis (which may be the individual patient, but may equally be a ward, unit, prescription, or antimicrobial episode in audit or interrupted time series [ITS] studies), and the professional implementer (the nurse or nursing team delivering the AMS task). A study is eligible where an AMS intervention delivered by, or involving, nursing staff is described and where the clinical population and/or unit of analysis relevant to that intervention is reported; studies are not required to report individual-level nurse characteristics where the unit of analysis is a ward, prescription, or episode, provided the nursing role in delivering the intervention is clearly described. Adult patients (18 years or older) receiving antimicrobial therapy in hospital inpatient settings (general wards, surgical wards, ICU, emergency departments with admitted patients, and infectious disease wards) or primary care and community settings are eligible. Eligible nurse populations include registered nurses, licensed practical/vocational nurses, nurse practitioners, advanced practice registered nurses, clinical nurse specialists, infection control nurses, ward nurses, primary/community nurses or nurse prescribers, and bedside nurses in any AMS-designated role. Pediatric-only settings and nursing home or long-term care facilities are excluded, for the reasons set out in the *Introduction* section.

#### Intervention (I)

A nurse-led or nurse-involved AMS intervention is any structured, planned program or formal protocol in which a nurse performs at least one clearly defined, nurse-initiated or nurse-performed AMS task, either independently, semiautonomously, or as the designated driver within a multidisciplinary team, classified according to the typology above. A nurse must have decision-making authority over, or protocol-driven initiation of, the AMS task; studies in which nurses serve only as passive data collectors, with no AMS-specific action attributable to them, do not meet this threshold. Eligible intervention types include intravenous-to-oral switch screening; antibiotic time-out led by nursing staff; culture-before-antibiotic protocol; patient and family education; allergy verification and de-labeling; antimicrobial prescribing audit and feedback; antimicrobial de-escalation support; medication reconciliation with AMS integration; and nurse-led AMS education program with outcome measurement; and sepsis bundle management with AMS component.

#### Comparator (C)

A formal comparison group is not required for inclusion. Where present, eligible comparators include standard care without a nursing AMS component; physician-led or pharmacist-led AMS without a designated nursing role; preintervention vs postintervention data; and control wards or hospitals without the nurse AMS program. Single-arm and uncontrolled before-and-after (UBA) studies will be considered for stream 1 narrative synthesis but will not be pooled in the primary quantitative meta-analysis and will not directly inform GRADE (Grading of Recommendations Assessment, Development and Evaluation) certainty ratings for pooled estimates; UBA studies will instead contribute contextual evidence to the stage C joint display. Where a GRADE-rated outcome draws on a body of evidence that includes UBA studies alongside controlled designs, this will be stated explicitly and the contribution of UBA data described narratively alongside the pooled estimate.

#### Outcomes (O)

##### Stream 1—Primary Quantitative Outcomes (Mandatory for Stream 1 Inclusion)

A study must report data for at least one of the following to be eligible for stream 1:

- Antibiotic consumption: days of therapy per 1000 patient-days (preferred), defined daily doses per 1000 patient-days, or total antibiotic units dispensed
- Appropriateness of antimicrobial prescribing: the proportion of prescriptions judged appropriate, appropriate with modification, or inappropriate against local, national, and international guidelines
- Clinical patient outcomes: all-cause in-hospital mortality, infection-attributed mortality, hospital length of stay in days, or ICU length of stay

The primary quantitative outcomes for stream 1 are described as follows:

- Time to first antibiotic dose: hours from clinical assessment or sepsis recognition to the first antibiotic administration—a directly nurse-influenced process outcome. Because clinically meaningful time frames for this outcome differ by setting (eg, emergency department sepsis pathways vs general ward review), studies will be grouped by care setting at analysis, and this heterogeneity discussed explicitly rather than pooled without adjustment where settings differ substantially.

- Rate of blood culture collection before first antibiotic dose: the proportion of eligible patients for whom microbiological specimens were obtained before the first antibiotic dose.

##### Stream 1–Secondary Quantitative Outcomes (Optional, Extracted Where Reported)

The following secondary quantitative outcomes will be extracted for stream 1 when reported by the included studies:

- *C. difficile* infection rate

- Health care–associated infection rates, including catheter-associated urinary tract infection, ventilator-associated pneumonia, and central line–associated bloodstream infection
- Multidrug-resistant organism isolation rates, including methicillin-resistant *Staphylococcus aureus*, carbapenem-resistant *Enterobacterales*, and extended-spectrum beta-lactamase–producing organisms

- Hospital readmission rates at 30 days (all-cause or infection-related)
- Antibiotic-related cost savings (direct drug costs; currency and reference year must be reported)
- Nurses’ compliance rate with the AMS program protocol

##### Stream 2–Implementation and Qualitative Outcomes (Mandatory for Stream 2 Inclusion)

A study must provide qualitative data, stakeholder perspectives, or contextual implementation information mappable to at least one CFIR 2.0 domain, including facilitators and barriers to nurse-led AMS implementation; nurse knowledge and clinical confidence before and after AMS training; stakeholder perceptions of the nurse’s AMS role; program fidelity; and program sustainability at 6 or 12 months post implementation.

### Study Design (S)

#### Stream 1–Quantitative Synthesis and Meta-Analysis

The following study designs will be included in the stream 1 quantitative synthesis and meta-analysis:

- Randomized controlled trials (RCTs) including cluster-randomized and stepped-wedge designs
- Quasi-experimental studies: non-RCTs with a concurrent or historical comparison group

- ITS: minimum 3 clearly defined measurement time points before and after the intervention
- Controlled before-and-after (CBA) studies
- UBA studies: narrative synthesis only; not pooled in primary meta-analysis

Handling of design-specific analytic issues will follow current methodological guidance. For pre-post and CBA studies, change scores will be used where reported; where only baseline and follow-up means are available, correlation coefficients from comparable studies will be imputed to estimate the SD of the change score, and any imputation will be reported and tested in sensitivity analysis; adjusted estimates will be preferred over unadjusted estimates where both are reported, and studies with missing SDs that cannot be estimated by any of these methods will be described narratively rather than pooled. For cluster-randomized and stepped-wedge trials, intracluster correlation coefficients (ICCs) and cluster-adjusted SEs will be extracted or, where not reported, estimated using a plausible ICC informed by comparable published cluster trials, with the source and value of any imputed ICC reported transparently.

- Minimum follow-up: given the differing clinical significance of the follow-up period across outcome types, the primary meta-analysis will require a minimum of 3 months of postintervention outcome measurement for multidrug-resistant organism and resistance-related outcomes, for which shorter windows are unlikely to capture meaningful change; for process outcomes such as time to the first antibiotic dose or blood culture collection rate, studies with shorter follow-up will also be eligible, since these outcomes can be meaningfully measured within a single admission or care episode, and follow-up duration will be reported and considered as a potential source of heterogeneity.

#### Stream 2–Qualitative/CFIR Implementation Narrative Synthesis

The following study designs will be included in the stream 2 qualitative and implementation synthesis:

- Qualitative studies employing any interpretive, descriptive, or mixed methodology
- Survey studies reporting stakeholder perceptions or clinical confidence levels
- No language restriction will be applied

Study design exclusions for both streams include the following:

- Case reports and case series
- Editorials, letters to the editors, expert opinion pieces, and commentaries without primary data
- Conference abstracts without sufficient extractable data

- Simulation studies assessing nurse AMS training only, without the measurement of clinical or behavioral outcomes in a real clinical setting

- Studies published before January 2000
  The January 2000 start date was selected because it corresponds to the period in which formal AMS as a defined, protocolized program model began to emerge internationally, ahead of the 2007 Infectious Diseases Society of America and the Society for Healthcare Epidemiology of America guidelines that established the current institutional standard [4,23]; earlier studies are unlikely to describe AMS interventions consistent with this review’s eligibility criteria, reflecting the rapid evolution of AMS frameworks post 2000. Within this window, the prespecified pre- and post-COVID-19 subgroup boundary of March 2020 marks the World Health Organization (WHO)’s declaration of the COVID-19 pandemic and the associated disruption to antimicrobial use, care delivery, and nursing workflows that followed.

### Information Sources and Search Strategy

An information scientist will develop and execute the final search strategy in collaboration with the lead author (SP), and all database-specific strings will be independently peer-reviewed prior to execution, with truncation and wildcard use (eg, “antibiotic optimi*”* and “nurse prescrib”) checked for consistency across databases to maximize sensitivity. The search will be reported in accordance with the PRISMA-S (Preferred Reporting Items for Systematic Reviews and Meta-Analyses—Search) extension for reporting literature searches in systematic reviews [33], which will also be mentioned in the completed review text with a corresponding reference. Searches will be conducted across 11 databases from January 2000 to the calendar month of searching: MEDLINE (PubMed), Embase (Ovid), CINAHL Complete (EBSCO), Cochrane CENTRAL (Cochrane Library), Scopus (Elsevier), Web of Science Core Collection (Clarivate), WHO Global Index Medicus, LILACS (VHL/BIREME), ClinicalTrials.gov, WHO International Clinical Trials Registry Platform, and Google Scholar (gray literature supplementary search). The search date, database version, and exact record yield for each database will be documented in a search log.

The search strategy uses six structured keyword blocks: Block A (nurses and nursing), Block B (AMS and AMS interventions), Block C (clinical outcomes, applied at full-text screening or to narrow a large yield), Block D (CFIR and implementation science, a supplementary qualitative search), Block E (health care settings), and Block F (GCC/MENA countries, for the subgroup supplementary search). Free-text terms are combined with controlled vocabulary (MeSH for MEDLINE, EMTREE for Embase, and CINAHL headings for CINAHL) using Boolean operators. The full database-specific search strings are provided in Multimedia Appendix 1.

Searches will be limited to English-language publications, with the exception that Arabic-language studies will also be retrieved and translated where GCC/MENA-relevant, given the review’s regional subgroup focus. This English-language restriction, common in systematic reviews but a recognized potential source of language bias, is a deliberate trade-off against the translation resources available to the team; it will be reported as a limitation. French-language publications are of particular relevance, given the concentration of Francophone health systems in North Africa (Morocco, Algeria, and Tunisia); where French-language full texts are identified during screening, they will be retained for translation and inclusion rather than excluded outright, and this will be recorded in the PRISMA 2020 [34] flow diagram (Multimedia Appendix 2) as a distinct exclusion/inclusion category. A dedicated Arabic-language database (eg, Dar Al-Mandumah) has not been added within the current database list, given licensing and access constraints; this is noted as a limitation and will be reconsidered if institutional access can be secured before searches commence.

Reference lists of all included systematic reviews and relevant primary studies will be hand-searched. Gray literature searches will include the WHO International Clinical Trials Registry Platform and ClinicalTrials.gov trial registries, together with a structured Google Scholar search. The Google Scholar search will use the Block A/B/E free-text terms combined with Boolean AND, sorted by relevance, with the first 200 results screened by title; the search date, exact query string, and number of results screened will be recorded in the search log, and all Google Scholar records identified as potentially eligible will be exported to Covidence for formal screening alongside database records.

### Study Selection

All retrieved records will be imported into Covidence and deduplicated. Title and abstract screening (round 1) will be performed independently by 2 reviewers, voting YES/MAYBE/NO based on the PICOS criteria. Any record receiving a YES or MAYBE vote from either reviewer will advance to full-text review. A Cohen κ coefficient of ≥0.70 is the prespecified threshold for acceptable interrater agreement at round 1. Where κ falls below this level, the team will reexamine the eligibility criteria and conduct a second calibration round on a further sample of records before proceeding; if agreement remains below 0.70 after this second round, a third senior reviewer will independently screen the full set of discordant records and adjudicate, and this contingency will be reported in the completed review regardless of whether it is invoked.

Full-text screening (round 2) will be performed independently by 2 reviewers against the complete inclusion and exclusion criteria. Reasons for exclusion will be recorded using a standardized exclusion code list in Covidence. All disagreements will be resolved through discussion; where consensus cannot be reached, a third senior reviewer will adjudicate. A PRISMA 2020 flow diagram will document record flow through all stages.

### Data Extraction

Two reviewers will conduct data extraction independently from all included studies using separate extraction forms for stream 1 (quantitative) and stream 2 (qualitative/CFIR-based), set up in Covidence. Prior to full extraction, both reviewers will pilot-extract data from the same randomly selected sample of 5 included studies. The 2 completed extraction sets will be compared at a dedicated team meeting; any discrepancies will be resolved and the extraction form revised accordingly before full extraction begins. Discrepancies during full extraction will be resolved at fortnightly team meetings; where agreement cannot be reached, a third senior reviewer will adjudicate. Because extraction fields differ substantially in structure, agreement will be quantified using a method matched to field type rather than a single global statistic. Cohen κ (target ≥0.70) will be calculated for categorical fields only (eg, study design classification, nursing-involvement typology, and CFIR domain coding); percentage agreement, supplemented by consensus discussion, will be used for continuous and free-text or verbatim fields (eg, effect estimates and verbatim nursing-role descriptions), for which a κ statistic is not appropriate. Where κ for a categorical field remains below 0.70 after pilot extraction, the relevant field definition will be revised and repiloted before full extraction proceeds; both pilot and full-extraction agreement statistics will be reported by field type in the completed review.

Quantitative extraction fields (stream 1) include study ID, author, year, country, setting; study design; sample size (patients and nurses, or ward/unit/prescription count where the unit of analysis is not the individual patient); nursing-involvement typology classification (see above); nurse role and AMS task description (verbatim from the paper); comparison/control group; primary and secondary outcomes measured with time points; results (means, medians, proportions, 95% CIs, and *P* values); GCC/MENA relevance (yes/no/partial). The full form is provided in Multimedia Appendix 3, which is the quantitative extraction form referenced throughout this paper.

Qualitative/CFIR extraction fields (stream 2) include study ID, country, setting, study method; participant roles; quotes and themes extracted and mapped to each of the 5 CFIR 2.0 domains; barrier/facilitator classification per domain; GCC/MENA relevance coding. The full form is provided in Multimedia Appendix 4.

### Quality Appraisal

Quality appraisal will be performed independently by 2 reviewers using tools matched to each study design, and disagreements will be resolved through discussion, with a third reviewer adjudicating where consensus is not reached. RCTs, including cluster-randomized designs, will be assessed with the Cochrane Risk of Bias 2 tool [35], which examines the randomization process, deviations from the intended intervention, missing outcome data, measurement of the outcome, and selection of reported results. Quasiexperimental and CBA studies will be assessed using ROBINS-I (Risk of Bias in Non-Randomized Studies of Interventions) [36], covering confounding, participant selection, classification of the intervention, deviations, missing data, outcome measurement, and reporting bias. ITS studies will be evaluated against the Cochrane Effective Practice and Organization of Care (EPOC) ITS criteria [37], which require that the intervention point was prespecified, that at least 3 measurement time points are available on each side of the intervention, that contemporaneous control data exist, and that outcome measurement is reliable. Qualitative studies will be appraised using the JBI Critical Appraisal Checklist for Qualitative Research [38], which is the appraisal tool recommended by JBI for use within the JBI mixed methods systematic review framework that guides this review. The checklist covers the clarity of research aims, appropriateness of methodology, recruitment strategy, data collection methods, researcher-participant relationship, ethical considerations, rigor of analysis, credibility of findings, and the study’s contribution to existing knowledge. Mixed methods studies will be assessed with the Mixed Methods Appraisal Tool [39], evaluating whether the mixed methods design is appropriate for the research question, whether the quantitative and qualitative components are adequately integrated, and whether divergence between components is addressed. Cross-sectional surveys will be appraised using the JBI Critical Appraisal Checklist for Analytical Cross-Sectional Studies [40], which covers sampling strategy, representativeness of the sample, validity of outcome measurement, response rate, and appropriateness of statistical analysis. The full set of quality-appraisal tools applied across study designs includes Cochrane Risk of Bias 2, ROBINS-I, the EPOC ITS criteria, the JBI qualitative checklist, Mixed Methods Appraisal Tool, and the JBI cross-sectional checklist. For nonrandomized studies appraised with ROBINS-I, GRADE certainty ratings will incorporate explicit downgrading for serious or critical risk of confounding bias, consistent with GRADE guidance for nonrandomized evidence, rather than being rated on the same starting basis as randomized evidence. Quality scores will not be used to exclude studies from the review but will directly inform GRADE certainty ratings and planned sensitivity analyses. Reviewers will not be masked to study authors or institutions during appraisal, given the practical constraints of a small review team; this is noted as a limitation. Appraisal results will be reported in narrative form and as summary tables in the final paper.

### Data Synthesis

#### Overview of the Synthesis Approach

Data analysis proceeds in 3 stages. Stage A addresses the quantitative effectiveness questions through statistical synthesis of stream 1 data. Stage B addresses the implementation and contextual questions through CFIR 2.0–informed qualitative synthesis of stream 2 data. Stage C integrates the outputs of stages A and B through a convergent joint display. The GCC/MENA subgroup will be addressed across all 3 stages.

#### Stage A: Quantitative Synthesis (Stream 1)

Where possible, outcomes will be converted to a common metric for pooling: risk ratios or odds ratios for dichotomous outcomes, mean differences or standardized mean differences for continuous outcomes reported on different scales, and rate ratios for consumption or infection-rate outcomes expressed as counts per patient-days. Outcomes that cannot be converted will be described narratively and excluded from statistical pooling for that specific outcome. Before any pooling is attempted, clinical and methodological homogeneity will be assessed by comparing populations, interventions, comparators, outcome measures, study designs, and follow-up periods.

The choice between fixed-effect and random-effects pooling will be made on conceptual grounds rather than on the basis of the *I*² statistic alone: given the anticipated diversity of nurse roles, health care settings, and patient populations across included studies, we consider it implausible that all studies estimate a single common effect, and a random-effects model will therefore be used for all pooled analyses as the prespecified default. Because the DerSimonian-Laird estimator can produce overly narrow CIs and inflated false-positive rates when the number of studies is small and heterogeneity is substantial, the Hartung-Knapp-Sidik-Jonkman method will be used as the primary variance estimator for all random-effects pooled estimates, with restricted maximum likelihood and conventional DerSimonian-Laird estimates reported as sensitivity analyses [41]. Because *I*² alone does not indicate the magnitude of variation in the true effect across settings, prediction intervals will be calculated and reported alongside each pooled estimate where a sufficient number of studies (generally 10 or more) contribute to that outcome to support the interpretation of the likely range of effect in an individual real-world setting rather than reliance on the average estimate alone [42]. Where *I*² exceeds 75% and the source of heterogeneity cannot be explained through prespecified subgroup analysis, pooling will be reconsidered for that outcome and findings reported narratively; where *I*² falls between 50% and 75%, the pooled estimate will be retained but heterogeneity will be explored through sensitivity analysis and the prespecified subgroup analyses. Meta-analysis will be performed using *R* (meta and metafor packages; R Foundation for Statistical Computing) and, where needed for cross-checking, Review Manager 5.4 (RevMan 5.4; Cochrane). Where meta-analysis is not feasible, a narrative synthesis will be conducted following the SWiM (synthesis without meta-analysis) reporting guideline [43].

Small-study effects will be assessed using funnel plots and the Egger regression test where 10 or more studies contribute to a pooled outcome. Funnel plot asymmetry and a significant Egger test will be interpreted as evidence of small-study effects, of which publication bias is one possible explanation among several (including genuine heterogeneity in study quality or true effect size by study size). This distinction will be stated explicitly wherever funnel plot results are reported [44,45].

The following 8 subgroup analyses are prespecified, provided that a minimum of 3 studies per subgroup report the outcome in question. To avoid overinterpretation of an excessive number of comparisons, 3 subgroups are designated as primary, confirmatory analyses that will be interpreted with standard significance thresholds and reported prominently, regardless of direction of findings; the remaining 5 are designated as exploratory and will be interpreted cautiously as hypothesis-generating, with results reported but not used to draw firm conclusions.

#### Primary Subgroups

The following 3 prespecified primary subgroup analyses will be conducted:

- Subgroup 1–region: GCC/MENA vs rest of the world
- Subgroup 2–clinical setting: hospital inpatient vs primary care or ambulatory

- Subgroup 3–level of nursing involvement: nurse-led vs nurse-initiated/semiautonomous vs nurse-involved with a defined AMS task, using the typology defined above

#### Exploratory subgroups

The following exploratory subgroup analyses will be conducted where sufficient data are available:

- Subgroup 4–ward type: ICU vs general medical or surgical wards
- Subgroup 5–intervention type: single-component vs multicomponent nurse AMS intervention
- Subgroup 6–country income level: high-income vs low- and middle-income countries (World Bank Classification)
- Subgroup 7–study design: RCTs only vs all study designs
- Subgroup 8–time period: pre-COVID-19 (before March 2020) vs post-COVID-19 (March 2020 onward)

#### Stage B: Qualitative and Implementation Synthesis (Stream 2)

Three reviewers, rather than 2, will independently code all included qualitative and mixed methods studies against the CFIR 2.0 framework, reducing the risk of subjective bias inherent in the dual coding of complex implementation data; discrepancies in domain or construct assignment will be resolved by discussion among the 3 coders, with a fourth senior reviewer adjudicating where consensus cannot be reached. All 3 reviewers will read included studies in full before extraction and coding begins. Each reviewer will independently extract data from all included studies into a CFIR 2.0 coding matrix. Relevant data include program context, barriers, facilitators, stakeholder perspectives, and implementation process descriptions, all mapped to the 5 CFIR 2.0 domains. Coding will use a hybrid deductive-inductive approach: data will first be deductively coded against the prespecified CFIR 2.0 domains and constructs, after which reviewers will inductively identify any recurring themes specific to nursing AMS roles that do not align cleanly with existing CFIR constructs, and these will be reported as a supplementary “nursing-specific” theme category alongside the CFIR-mapped findings. Following initial coding, thematic synthesis will be applied using the approach described by Thomas and Harden [46]: (1) free line-by-line coding of primary findings, (2) development of descriptive themes within each CFIR domain, and (3) the generation of analytic themes explaining patterns of implementation success or failure across studies. Barriers and facilitators will be classified within each domain, and findings will be presented as a domain-by-domain narrative summary accompanied by a structured CFIR matrix table.

#### Stage C: Convergent Integration Joint Display

Stage C will integrate the outputs of stages A and B using a convergent joint display [47]. The joint display is a structured matrix in which each row represents a CFIR 2.0 domain or construct and the columns represent (1) implementation facilitators and barriers identified in the qualitative synthesis; (2) corresponding quantitative outcomes and effect estimates from the meta-analysis; and (3) an integrated inference classified explicitly as convergence (both streams support the same conclusion), complementarity (streams address different but compatible aspects of the same domain), expansion (one stream extends or explains a finding from the other), or discordance (streams reach conflicting conclusions). Where discordance is identified, this will be reported transparently rather than resolved by preferring one stream over the other, and possible explanations for the discordance (eg, differing study contexts or outcome definitions) will be discussed; we acknowledge that the qualitative and quantitative streams may not always align neatly, and the integration approach is designed to surface, not obscure, such divergence. Where integration is not feasible for a given domain due to insufficient data in one stream, this will be reported transparently and both streams presented independently.

### GRADE Evidence Certainty Rating

The GRADE approach [48] will be applied to each primary quantitative outcome, following the completion of meta-analysis or narrative synthesis. Each outcome will be rated as high, moderate, low, or very low certainty by assessing five domains: (1) risk of bias, as determined by the quality appraisal tools described above, with explicit downgrading for confounding in ROBINS-I-appraised nonrandomized evidence as described under the *Quality Appraisal* section; (2) inconsistency, the degree of unexplained heterogeneity quantified by *I*² and interpreted alongside the prediction interval; (3) indirectness, whether included studies directly address the PICOS criteria; (4) imprecision, whether CIs around pooled estimates are wide; and (5) publication bias, assessed through funnel plot analysis where 10 or more studies contribute to a pooled estimate. GRADE summary of findings tables will be produced using GRADEpro GDT (McMaster University and Evidence Prime) and will be included in the final review manuscript.

### Reporting

PRISMA 2020 [34] will guide write-up of the completed review, supplemented by the PRISMA-CI (Preferred Reporting Items for Systematic Reviews and Meta-Analyses—Extension for Complex Interventions) [49] to support transparent reporting of the multicomponent AMS interventions and the CFIR-based contextual synthesis and by PRISMA-S [33] for the search strategy. Where meta-analysis is not feasible for one or more outcomes, the SWiM reporting guideline will be applied [43].

### Amendments

Any amendments made to this protocol after PROSPERO registration will be documented in PROSPERO, dated, and accompanied by a brief rationale. A summary table of all amendments, or a statement that none occurred, will be included in the completed review paper, consistent with the PRISMA-P 2015 [31] checklist requirement to report protocol amendments.

## Results

This protocol was registered on PROSPERO in March 2026 (registration number CRD420261341653). Database searches across all planned databases and sources, Covidence import, and deduplication were completed in March 2026. Title and abstract screening, including pilot calibration between reviewers, was completed by June 2026. Full-text screening against the complete eligibility criteria is planned to be completed by August 2026. Quantitative data extraction (stream 1) and qualitative CFIR data extraction (stream 2) are planned to run in parallel from August to September 2026. Meta-analysis (stage A), CFIR thematic synthesis (stage B), and GRADE evidence rating are anticipated to be completed by November 2026. Mixed methods integration (stage C, joint display) is planned for November to December 2026. Manuscript writing is planned for January to February 2027, with submission of the completed review targeted for March 2027. No amendments to the registered protocol have occurred. The projected timeline and status of each phase as of this protocol’s submission are summarized in Table 1.

**Table 1.** Projected timeline and status for the nurse-led antimicrobial stewardship mixed methods systematic review.

Timeline	Activity	Status
February to March 2026	Protocol finalization and PROSPERO^a^ registration	Completed
March 2026	Database searches executed across all planned databases and sources; search log maintained	Completed
March 2026	Records imported into Covidence; automatic and manual deduplication	Completed
April to June 2026	Title and abstract screening, including pilot calibration between reviewers	Completed
June to July 2026	Backward citation chasing and full-text retrieval of potentially eligible studies	Completed
July to August 2026	Full-text screening against the complete eligibility criteria	Planned
August to September 2026	Quantitative data extraction and quality appraisal (stream 1) and qualitative/CFIR^b^ data extraction and quality appraisal (stream 2), conducted in parallel	Planned
October 2026	Data harmonization and cleaning	Planned
October to November 2026	Meta-analysis and quantitative synthesis (stage A); CFIR thematic synthesis (stage B); GRADE^c^ evidence rating	Planned
November to December 2026	Mixed methods integration and joint display (stage C)	Planned
January to February 2027	Manuscript writing	Planned
March 2027	Internal review, final revision, and journal submission of the completed review	Planned

a PROSPERO: International Prospective Register of Systematic Reviews.

b CFIR: Consolidated Framework for Implementation Research.

c GRADE: Grading of Recommendations Assessment, Development and Evaluation.

## Discussion

### Principal Findings

This protocol describes what we believe, to the best of our knowledge, will be among the first systematic reviews to combine meta-analytic synthesis of nurse-led and nurse-involved AMS clinical effectiveness with a CFIR 2.0-informed implementation analysis and a prespecified GCC/MENA subgroup. A review of existing systematic reviews and protocols in this area confirms that each addresses only part of the evidence landscape: some measure knowledge but not outcomes, some apply qualitative synthesis but not meta-analysis, and none has used CFIR 2.0 to explain implementation variation or produced regional estimates for GCC/MENA settings.

### Comparison With Prior Work

A thorough search of PROSPERO, the Cochrane Database of Systematic Reviews, Epistemonikos, and key databases (with searches completed in February 2026) identified 6 existing systematic reviews or registered protocols relevant to nursing and AMS. The PROSPERO-registered review by Blackburn et al [21] on nurses’ knowledge and perceptions (CRD42024524162, published in 2025) measured no clinical outcomes and was entirely scoped to attitudinal and perceptual data. A qualitative meta-synthesis by Bonacaro et al [22] (CRD42023460278, published in 2024) synthesized nurses’ experiences and barriers in stewardship across 19 qualitative studies (2018‐2023) but did not attempt meta-analysis, apply an implementation science framework, or include a GCC/MENA subgroup. The NREM-based mapping review by Filipe et al (OSF-registered) [20] found that only 9% of included studies reported clinical outcome data and explicitly excluded those studies from its scope. A systematic review of ASP implementation in Middle Eastern countries by Ababneh et al [25] covered the regional literature but included only physician-led and pharmacist-led programs, with searches ending in December 2020. A CFIR-guided review of ASP implementation in sub-Saharan Africa by Kapatsa et al [28] applied CFIR to a geographically distinct scope and was not nursing-specific. Finally, a global umbrella review of ASP effectiveness by Krishnamoorthy et al (CRD42024541821) [50] covered all ASP types but was not specific to nursing and noted GCC/MENA as a gap area. None of these reviews simultaneously combines nurse-led clinical effectiveness with meta-analysis, a CFIR 2.0 implementation synthesis, a prespecified GCC/MENA subgroup, and PROSPERO registration. The present review is intended to integrate all 4 components. We will update this comparison before submission of the completed review to capture any reviews published during the conduct of this study.

### Limitations

Several limitations are anticipated. First, clinical heterogeneity across included studies is expected, given differences in nurse AMS roles, hospital organizational structures, antimicrobial prescribing cultures, and national AMR policy environments. This may limit the feasibility of pooling for some outcomes, in which case narrative synthesis and prespecified subgroup analyses will be used, and prediction intervals will be reported to support the interpretation of likely real-world variation in effect. Second, the GCC/MENA subgroup may be constrained by small study numbers; finding few or even no eligible studies in this region would itself be a substantive and publishable finding, highlighting a clear research gap, though the validity and interpretability of any subgroup comparison based on a very small evidence base will need to be discussed cautiously in the completed review. Third, publication bias is a recognized limitation of meta-analysis; as noted under the *Data Synthesis* section, funnel plots and the Egger test will be used to assess small-study effects, of which publication bias is one possible cause. Fourth, restricting searches primarily to English-language literature (with the exception of Arabic-language studies retrieved for GCC/MENA relevance) risks omitting relevant evidence, particularly French-language literature from North African health systems; the scale of this potential bias will be assessed as described under the *Information Sources and Search Strategy* section. Fifth, heterogeneity in nurse education and scope of practice (diploma, degree, and nurse practitioner level) across included studies is anticipated and will be extracted and reported descriptively, though it may not be possible to fully adjust pooled estimates for this factor. Sixth, because some studies may contribute data to both the quantitative and qualitative streams, the risk of the same underlying dataset being counted more than once in the mixed methods integration will be assessed by cross-checking study author groups, institutions, and recruitment periods at the data extraction stage. Seventh, the joint display integration depends on both streams yielding sufficient data; where one stream is sparse, findings will be presented independently rather than forced into an integration that the data cannot support. Finally, this protocol does not report a formal mechanism for patient or public involvement in its design, which is an increasingly expected feature of systematic review conduct; this is acknowledged as a limitation. No mechanism is currently planned for updating the review, should major new trials be published during the analysis window; any update would be considered as a distinct future project rather than a protocol amendment.

### Conclusions

Findings from this review are intended to inform (1) evidence-based hospital ASP design, by identifying which nurse-led AMS tasks produce the strongest clinical signals; (2) nursing education and training curricula, by identifying the knowledge and skill gaps that most consistently function as implementation barriers; (3) health workforce policy in GCC/MENA settings, where this review is intended to provide one of the first regional evidence syntheses on nursing AMS roles, given the anticipated scarcity of GCC/MENA-specific studies and the corresponding need for cautious interpretation of any subgroup findings from this region; and (4) future primary research priorities, by mapping the gaps that even a thorough synthesis cannot fill.

## Supplementary material

10.2196/96342 Multimedia Appendix 1 Search strings—all databases.

10.2196/96342 Multimedia Appendix 2 PRISMA (Preferred Reporting Items for Systematic Reviews and Meta-Analyses) 2020 flow diagram.

10.2196/96342 Multimedia Appendix 3 Quantitative data extraction form.

10.2196/96342 Multimedia Appendix 4 Qualitative data extraction form.

10.2196/96342 Checklist 1 PRISMA-P checklist.

## Abbreviations

AMR: antimicrobial resistance
AMS: antimicrobial stewardship
ASP: antimicrobial stewardship program
CBA: controlled before-and-after
CFIR: Consolidated Framework for Implementation Research
CRE: carbapenem-resistant *Enterobacterales*
EPOC: Effective Practice and Organization of Care
GCC: Gulf Cooperation Council
GRADE: Grading of Recommendations Assessment, Development and Evaluation
ICC: intracluster correlation coefficient
ICU: intensive care unit
ITS: interrupted time series
JBI: Joanna Briggs Institute
MENA: Middle East and North Africa
NREM: Nursing Role Effectiveness Model
PICOS: Population, Intervention, Comparator, Outcomes, Study design
PRISMA: Preferred Reporting Items for Systematic Reviews and Meta-Analyses
PRISMA-CI: Preferred Reporting Items for Systematic Reviews and Meta-Analyses—Extension for Complex Interventions
PRISMA-P: Preferred Reporting Items for Systematic Reviews and Meta-Analyses—Extension for Protocols
PRISMA-S: Preferred Reporting Items for Systematic Reviews and Meta-Analyses—Extension for Reporting Literature Searches
PROSPERO: International Prospective Register of Systematic Reviews
RCT: randomized controlled trial
ROBINS-I: Risk Of Bias In Non-randomized Studies of Interventions
SWiM: synthesis without meta-analysis
UBA: uncontrolled before-and-after
WHO: World Health Organization
