# Supplementary material for: Nurse-Led and Nurse-Involved Antimicrobial Stewardship in Hospitals and Primary Care: Protocol for a Mixed Methods Systematic Review and Meta-Analysis With Gulf Cooperation Council and Middle East and North Africa Subgroup Analysis
Source: JMIR Res Protoc. 2026 Oct 8;15:e96342. doi: 10.2196/96342 (PMC13649170; doi:10.2196/96342)
Supplement: Supplementary file 1 [file resprot-v15-e96342-s005.pdf]

Multimedia Appendix 4. PRISMA-P 2015 Checklist

This checklist has been adapted for use with protocol submissions to *Systematic Reviews* from Table 3 in Moher D et al: Preferred reporting items for systematic review and meta-analysis protocols (PRISMA-P) 2015 statement. *Systematic Reviews* 2015 **4**:1

Section/topic	#	Checklist item	Information reported		Line number(s)
			Yes	No	
**ADMINISTRATIVE INFORMATION**					
**Title**					
Identification	1a	Identify the report as a protocol of a systematic review	Yes		Title; p.1
Update	1b	If the protocol is for an update of a previous systematic review, identify as such		No (N/A – new protocol)	
**Registration**	2	If registered, provide the name of the registry (e.g., PROSPERO) and registration number in the Abstract	Yes		p.2 (Trial Registration); p.17 (Results)
**Authors**					
Contact	3a	Provide name, institutional affiliation, and e-mail address of all protocol authors; provide physical mailing address of corresponding author	Yes		Title page
Contributions	3b	Describe contributions of protocol authors and identify the guarantor of the review	Yes		p.21 (Authors' Contributions)
**Amendments**	4	If the protocol represents an amendment of a previously completed or published protocol, identify as such and list changes; otherwise, state plan for documenting important protocol amendments	Yes		p.17 (Methods, Amendments); p.17 (Results)
**Support**					
Sources	5a	Indicate sources of financial or other support for the review	Yes		p.20 (Funding)
Sponsor	5b	Provide name for the review funder and/or sponsor	Yes		p.20 (Funding)
Role of sponsor/funder	5c	Describe roles of funder(s), sponsor(s), and/or institution(s), if any, in developing the protocol	Yes		p.20 (Funding)
**INTRODUCTION**					

BioMed Central
The Open Access Publisher

Section/topic	#	Checklist item	Information reported		Line number(s)
			Yes	No	
**Rationale**	6	Describe the rationale for the review in the context of what is already known	Yes		p.2–5 (Introduction)
**Objectives**	7	Provide an explicit statement of the question(s) the review will address with reference to participants, interventions, comparators, and outcomes (PICO)	Yes		p.1 (Abstract, Objective); p.5 (Research Questions)
**METHODS**					
**Eligibility criteria**	8	Specify the study characteristics (e.g., PICO, study design, setting, time frame) and report characteristics (e.g., years considered, language, publication status) to be used as criteria for eligibility for the review	Yes		p.7–11 (Eligibility Criteria)
**Information sources**	9	Describe all intended information sources (e.g., electronic databases, contact with study authors, trial registers, or other grey literature sources) with planned dates of coverage	Yes		p.11–12 (Information Sources and Search Strategy)
**Search strategy**	10	Present draft of search strategy to be used for at least one electronic database, including planned limits, such that it could be repeated	Yes		p.11–12; full strings in Multimedia Appendix 1
STUDY RECORDS					
Data management	11a	Describe the mechanism(s) that will be used to manage records and data throughout the review	Yes		p.12 (Study Selection)
Selection process	11b	State the process that will be used for selecting studies (e.g., two independent reviewers) through each phase of the review (i.e., screening, eligibility, and inclusion in meta-analysis)	Yes		p.12 (Study Selection)
Data collection process	11c	Describe planned method of extracting data from reports (e.g., piloting forms, done independently, in duplicate), any processes for obtaining and confirming data from investigators	Yes		p.12–13 (Data Extraction)
**Data items**	12	List and define all variables for which data will be sought (e.g., PICO items, funding sources), any pre-planned data assumptions and simplifications	Yes		p.12–13 (Data Extraction)
**Outcomes and prioritization**	13	List and define all outcomes for which data will be sought, including prioritization of main and additional outcomes, with rationale	Yes		p.8–9 (Eligibility Criteria, Outcomes)

Section/topic	#	Checklist item	Information reported		Line number(s)
			Yes	No	
**Risk of bias in individual studies**	14	Describe anticipated methods for assessing risk of bias of individual studies, including whether this will be done at the outcome or study level, or both; state how this information will be used in data synthesis	Yes		p.13–14 (Quality Appraisal)
DATA					
**Synthesis**	15a	Describe criteria under which study data will be quantitatively synthesized	Yes		p.14–15 (Data Synthesis, Stage A)
	15b	If data are appropriate for quantitative synthesis, describe planned summary measures, methods of handling data, and methods of combining data from studies, including any planned exploration of consistency (e.g., I^2, Kendall's tau)	Yes		p.14–15 (Data Synthesis, Stage A)
	15c	Describe any proposed additional analyses (e.g., sensitivity or subgroup analyses, meta-regression)	Yes		p.14–15 (Data Synthesis, Stage A)
	15d	If quantitative synthesis is not appropriate, describe the type of summary planned	Yes		p.15 (Data Synthesis, Stage A – SWiM)
**Meta-bias(es)**	16	Specify any planned assessment of meta-bias(es) (e.g., publication bias across studies, selective reporting within studies)	Yes		p.15 (Data Synthesis, Stage A)
**Confidence in cumulative evidence**	17	Describe how the strength of the body of evidence will be assessed (e.g., GRADE)	Yes		p.16 (GRADE Evidence Certainty Rating)

BioMed Central
The Open Access Publisher
