# Supplementary material for: Nurse-Led and Nurse-Involved Antimicrobial Stewardship in Hospitals and Primary Care: Protocol for a Mixed Methods Systematic Review and Meta-Analysis With Gulf Cooperation Council and Middle East and North Africa Subgroup Analysis
Source: JMIR Res Protoc. 2026 Oct 8;15:e96342. doi: 10.2196/96342 (PMC13649170; doi:10.2196/96342)
Supplement: Supplementary file 2 [file resprot-v15-e96342-s001.docx]

**Multimedia Appendix 1 – Search Strings**

**1. Master Keyword Blocks**

The following blocks form the basis for all database-specific strings. Database-specific field codes and controlled vocabulary are added in each section below.

**BLOCK A — Nurses and Nursing (connect all terms with OR)**

**Free-text terms:**

nurs*

"nurse-led" OR "nurse led"

"nurse-driven" OR "nurse driven"

"nurse-initiated" OR "nurse initiated"

"registered nurse" OR "registered nurses"

"nurse practitioner" OR "nurse practitioners"

"advanced practice nurse" OR "advanced practice nursing"

"clinical nurse specialist" OR "clinical nurse specialists"

"nursing staff" OR "nursing personnel"

"bedside nurse*" OR "ward nurse*"

"ICU nurse*" OR "critical care nurse*"

"infection control nurse*" OR "infection prevention nurse*"

"primary care nurse*" OR "community nurse*"

▶ "nurse prescrib*"

▶ "advanced nurse practitioner*"

**Controlled vocabulary (MeSH/EMTREE/CINAHL):** Nurses | Nursing Staff, Hospital | Nurse Practitioners | Advanced Practice Nursing | Clinical Nurse Specialists

**BLOCK B — Antimicrobial Stewardship and Interventions (connect all terms with OR)**

**Free-text terms:**

**Controlled vocabulary:** MeSH: Antimicrobial Stewardship | Anti-Bacterial Agents (use with caution — combine with Block A nurse terms)

"antimicrobial stewardship" OR "antibiotic stewardship"

"antimicrobial stewardship program*" OR "antibiotic stewardship program*"

"antibiotic management" OR "antimicrobial management"

"rational antibiotic use" OR "appropriate antibiotic use" OR "appropriate antimicrobial use"

"antibiotic optimis*" OR "antibiotic optimiz*" OR "antimicrobial optimis*" OR "antimicrobial optimiz*"

"antibiotic timeout*" OR "antimicrobial timeout*" OR "antibiotic time-out*"

"IV to oral switch" OR "IV-to-oral" OR "IV-to-PO" OR "intravenous to oral" OR "parenteral to oral"

"de-escalation" AND (antibiotic* OR antimicrobial*)

"antibiotic de-escalation" OR "antimicrobial de-escalation"

"prospective audit and feedback" OR "antimicrobial audit" OR "antibiotic audit"

"antibiotic allergy" OR "penicillin allergy"

▶ "allergy de-labelling" OR "allergy delabelling"

▶ "penicillin de-labelling" OR "penicillin delabelling"

"antibiotic prescribing" OR "antimicrobial prescribing"

"formulary restriction" OR "prior authoris*" OR "prior authoriz*"

"medication reconciliation" AND (antibiotic* OR antimicrobial*)

▶ "sepsis bundle" AND (nurs* OR antibiotic* OR antimicrobial*)

▶ "blood culture" AND (nurs* OR compliance OR collection OR rate)

▶ "antibiotic review" OR "antimicrobial review"

▶ "antibiotic stewardship" AND (role* OR task*)

**BLOCK C — Clinical Outcomes (optional — apply only if primary yield exceeds 5,000 after deduplication)**

"antibiotic consumption" OR "antimicrobial consumption"

"days of therapy" OR "defined daily dose*"

"prescribing appropriateness" OR "appropriate prescribing"

mortality OR "all-cause mortality" OR "infection-related mortality"

"length of stay" OR "hospital stay"

"Clostridioides difficile" OR "Clostridium difficile" OR CDI

"healthcare-associated infection*" OR "hospital-acquired infection*" OR HAI

"multidrug-resistant" OR MDRO

"time to first dose" OR "time to antibiotic" OR "antibiotic timing"

"blood culture" AND (compliance OR collection OR rate)

**BLOCK D — CFIR and Implementation Science (supplementary qualitative/implementation search)**

"implementation science" OR "implementation research"

"Consolidated Framework for Implementation Research" OR CFIR OR "CFIR 2.0"

"barriers" AND (AMS OR "antibiotic stewardship" OR "antimicrobial stewardship" OR nurs*)

"facilitators" AND (AMS OR "antibiotic stewardship" OR "antimicrobial stewardship" OR nurs*)

"implementation barriers" OR "implementation facilitators" OR "implementation determinants"

"implementation fidelity" OR "programme fidelity" OR "intervention fidelity"

"Normalisation Process Theory" OR NPT

"knowledge translation" OR "evidence-based practice implementation"

"organisational readiness" OR "organizational readiness" OR "implementation readiness"

interview* OR "focus group*" OR "qualitative stud*" OR "stakeholder perception*"

**BLOCK E — Healthcare Settings (apply where needed to filter by setting)**

hospital* OR inpatient* OR "acute care"

"intensive care unit" OR ICU OR "critical care"

"surgical ward*" OR "medical ward*" OR "general ward*"

"primary care" OR "community health" OR outpatient*

"general practice" OR "family medicine" OR "family practice"

"emergency department" OR "emergency medicine"

**BLOCK F — GCC/MENA Countries (subgroup analysis supplementary search)**

"Gulf Cooperation Council" OR GCC

"Middle East and North Africa" OR MENA OR "Eastern Mediterranean"

"Saudi Arabia" OR "Kingdom of Saudi Arabia" OR KSA

"United Arab Emirates" OR UAE OR "Abu Dhabi" OR Dubai

Qatar OR Kuwait OR Bahrain OR Oman

Jordan OR Lebanon OR Egypt OR Syria OR Iraq OR Iran

Morocco OR Tunisia OR Algeria OR Libya OR Sudan OR Yemen

"Palestine" OR "Palestinian territories"

"Arab world" OR "Arab countries" OR "Arab states" OR "WHO EMRO"

**2. Search Strings – All Databases**

**Database 1: MEDLINE via PubMed**

Search 1: Primary (Block A AND Block B)

(nurs*[tiab] OR "nurse-led"[tiab] OR "nurse led"[tiab] | | OR "nurse-driven"[tiab] OR "nurse driven"[tiab] | | OR "nurse-initiated"[tiab] OR "nurse initiated"[tiab] | | OR "registered nurse"[tiab] OR "registered nurses"[tiab] | | OR "nurse practitioner"[tiab] OR "nurse practitioners"[tiab] | | OR "advanced practice nurse"[tiab] OR "advanced practice | nursing"[tiab] | | OR "clinical nurse specialist"[tiab] OR "clinical nurse | specialists"[tiab] | | OR "nursing staff"[tiab] OR "nursing personnel"[tiab] | | OR "bedside nurse"[tiab] OR "bedside nurses"[tiab] | | OR "ward nurse"[tiab] OR "ward nurses"[tiab] | | OR "ICU nurse"[tiab] OR "ICU nurses"[tiab] | | OR "critical care nurse"[tiab] OR "critical care | nurses"[tiab] | | OR "infection control nurse"[tiab] OR "infection control | nurses"[tiab] | | OR "infection prevention nurse"[tiab] OR "infection prevention | nurses"[tiab] | | OR "primary care nurse"[tiab] OR "primary care nurses"[tiab] | | OR "community nurse"[tiab] OR "community nurses"[tiab] | | OR "nurse prescrib*"[tiab] | | OR "advanced nurse practitioner"[tiab] OR "advanced nurse | practitioners"[tiab] | | OR Nurses[MeSH] OR "Nursing Staff, Hospital"[MeSH] | | OR "Nurse Practitioners"[MeSH] OR "Advanced Practice | Nursing"[MeSH] | | OR "Clinical Nurse Specialists"[MeSH]) | | AND | | ("antimicrobial stewardship"[tiab] OR "antibiotic | stewardship"[tiab] | | OR "antimicrobial stewardship program"[tiab] OR "antimicrobial | stewardship programme"[tiab] | | OR "antibiotic stewardship program"[tiab] OR "antibiotic | stewardship programme"[tiab] | | OR "antibiotic management"[tiab] OR "antimicrobial | management"[tiab] | | OR "rational antibiotic use"[tiab] OR "appropriate antibiotic | use"[tiab] | | OR "appropriate antimicrobial use"[tiab] | | OR "antibiotic optimis*"[tiab] OR "antibiotic optimiz*"[tiab] | OR "antimicrobial optimis*"[tiab] OR "antimicrobial | optimiz*"[tiab] | | OR "antibiotic timeout"[tiab] OR "antibiotic time-out"[tiab] | | OR "antimicrobial timeout"[tiab] OR "antimicrobial | time-out"[tiab] | | OR "IV-to-oral"[tiab] OR "IV-to-PO"[tiab] | | OR "intravenous to oral"[tiab] OR "parenteral to oral"[tiab] | | OR "de-escalation"[tiab] OR "antibiotic de-escalation"[tiab] | | OR "antimicrobial de-escalation"[tiab] | | OR "prospective audit and feedback"[tiab] OR "antimicrobial | audit"[tiab] | | OR "antibiotic audit"[tiab] | | OR "antibiotic allergy"[tiab] OR "penicillin allergy"[tiab] | | OR "allergy de-labelling"[tiab] OR "allergy | delabelling"[tiab] | | OR "penicillin de-labelling"[tiab] OR "penicillin | delabelling"[tiab] | | OR "antibiotic prescribing"[tiab] OR "antimicrobial | prescribing"[tiab] | | OR "formulary restriction"[tiab] | | OR "prior authorisation"[tiab] OR "prior authorization"[tiab] | | OR "sepsis bundle"[tiab] | | OR "antibiotic review"[tiab] OR "antimicrobial review"[tiab] | | OR "Antimicrobial Stewardship"[MeSH] OR "Anti-Bacterial | Agents"[MeSH]) | | AND ("2000/01/01"[PDAT] : "3000/12/31"[PDAT])

Search 2: CFIR/Implementation Supplement (Block A AND Block B AND Block D)

AND

("implementation science"[tiab] OR "implementation research"[tiab]

OR "Consolidated Framework for Implementation Research"[tiab]

OR CFIR[tiab] OR "CFIR 2.0"[tiab]

OR "implementation barriers"[tiab] OR "implementation facilitators"[tiab]

OR "implementation determinants"[tiab] OR "implementation fidelity"[tiab]

OR "Normalisation Process Theory"[tiab] OR NPT[tiab]

OR "knowledge translation"[tiab]

OR "organisational readiness"[tiab] OR "organizational readiness"[tiab]

OR interview*[tiab] OR "focus group"[tiab] OR "focus groups"[tiab]

OR "qualitative stud"[tiab] OR "stakeholder perception"[tiab])

Search 3: GCC/MENA Supplement (Block A AND Block B AND Block F)

AND

("Gulf Cooperation Council"[tiab] OR GCC[tiab]

OR "Middle East and North Africa"[tiab] OR MENA[tiab]

OR "Eastern Mediterranean"[tiab]

OR "Saudi Arabia"[tiab] OR "Kingdom of Saudi Arabia"[tiab] OR KSA[tiab]

OR "United Arab Emirates"[tiab] OR UAE[tiab] OR "Abu Dhabi"[tiab] OR Dubai[tiab]

OR Qatar[tiab] OR Kuwait[tiab] OR Bahrain[tiab] OR Oman[tiab]

OR Jordan[tiab] OR Lebanon[tiab] OR Egypt[tiab] OR Syria[tiab]

OR Iraq[tiab] OR Iran[tiab] OR Morocco[tiab] OR Tunisia[tiab]

OR Algeria[tiab] OR Libya[tiab] OR Sudan[tiab] OR Yemen[tiab]

OR "Arab world"[tiab] OR "Arab countries"[tiab] OR "WHO EMRO"[tiab])

**Database 2: EMBASE via Ovid**

Search Set 1: Primary (Lines 1–9)

1. (nurs* OR "nurse-led" OR "nurse led" OR "nurse-driven" OR | "nurse driven" | | OR "nurse-initiated" OR "nurse initiated" OR "registered nurse" | | OR "registered nurses" OR "nurse practitioner" OR "nurse | practitioners" | | OR "advanced practice nurse" OR "advanced practice nursing" | | OR "clinical nurse specialist" OR "clinical nurse specialists" | | OR "nursing staff" OR "nursing personnel" OR "bedside nurse*" | | OR "ward nurse*" OR "ICU nurse*" OR "critical care nurse*" | | OR "infection control nurse*" OR "infection prevention nurse*" | | OR "primary care nurse*" OR "community nurse*" | | OR "nurse prescrib*" OR "advanced nurse practitioner*").ti,ab. | | 2. exp nurse/ OR exp nursing staff/ OR exp nurse practitioner/ | | OR exp advanced practice nurse/ OR exp clinical nurse specialist/ | | 3. 1 OR 2 | | 4. ("antimicrobial stewardship" OR "antibiotic stewardship" | | OR "antimicrobial stewardship program*" OR "antibiotic | stewardship program*" | | OR "antibiotic management" OR "antimicrobial management" | | OR "rational antibiotic use" OR "appropriate antibiotic use" | | OR "appropriate antimicrobial use" OR "antibiotic optimi*" | | OR "antimicrobial optimi*" OR "antibiotic timeout" OR "antibiotic time-out" | | OR "antimicrobial timeout" OR "IV-to-oral" OR "IV-to-PO" | | OR "intravenous to oral" OR "parenteral to oral" | | OR "de-escalation" OR "antibiotic de-escalation" OR | "antimicrobial de-escalation" | | OR "prospective audit and feedback" OR "antimicrobial audit" OR | "antibiotic audit" | | OR "antibiotic allergy" OR "penicillin allergy" | | OR "allergy de-labelling" OR "allergy delabelling" | | OR "penicillin de-labelling" OR "penicillin delabelling" | | OR "antibiotic prescribing" OR "antimicrobial prescribing" | | OR "formulary restriction" OR "prior authoris*" OR "prior | authoriz*" | | OR "sepsis bundle" OR "antibiotic review" OR "antimicrobial | review" | | OR "medication reconciliation").ti,ab. | | 5. exp antimicrobial stewardship/ OR exp antibiotic agent/ | | OR exp antibiotic therapy/ OR exp antibiotic prophylaxis/ | | 6. 4 OR 5 | | 7. 3 AND 6 | | 8. limit 7 to yr="2000-Current" | | 9. limit 8 to (article or review or "systematic review" or | conference paper)

Search Set 2: CFIR Supplement (Lines 10–14)

10. ("implementation science" OR "implementation research"

OR "Consolidated Framework for Implementation Research"

OR CFIR OR "CFIR 2.0" OR "implementation barriers"

OR "implementation facilitators" OR "implementation determinants"

OR "implementation fidelity" OR "Normalisation Process Theory" OR NPT

OR "knowledge translation" OR "organisational readiness"

OR "organizational readiness" OR interview* OR "focus group*"

OR "qualitative stud*" OR "stakeholder perception*").ti,ab.

11. exp implementation science/ OR exp qualitative research/

OR exp focus group/ OR exp interview/

12. 10 OR 11

13. 7 AND 12

14. limit 13 to yr="2000-Current"

Search Set 3: GCC/MENA Supplement (Lines 15–17)

15. ("Gulf Cooperation Council" OR GCC

OR "Middle East and North Africa" OR MENA OR "Eastern Mediterranean"

OR "Saudi Arabia" OR "Kingdom of Saudi Arabia" OR KSA

OR "United Arab Emirates" OR UAE OR "Abu Dhabi" OR Dubai

OR Qatar OR Kuwait OR Bahrain OR Oman

OR Jordan OR Lebanon OR Egypt OR Syria

OR Iraq OR Iran OR Morocco OR Tunisia

OR Algeria OR Libya OR Sudan OR Yemen

OR "Arab world" OR "Arab countries" OR "WHO EMRO").ti,ab.

16. 7 AND 15

17. limit 16 to yr="2000-Current"

**Database 3: CINAHL Complete via EBSCO**

Search 1: Primary (Block A AND Block B)

( TI(nurs* OR "nurse-led" OR "nurse led" OR "nurse-driven"

OR "nurse-initiated" OR "registered nurse" OR "registered nurses"

OR "nurse practitioner" OR "nurse practitioners"

OR "advanced practice nurse" OR "advanced practice nursing"

OR "clinical nurse specialist" OR "clinical nurse specialists"

OR "nursing staff" OR "nursing personnel" OR "bedside nurse*"

OR "ward nurse*" OR "ICU nurse*" OR "critical care nurse*"

OR "infection control nurse*" OR "infection prevention nurse*"

OR "primary care nurse*" OR "community nurse*"

OR "nurse prescrib*" OR "advanced nurse practitioner*")

OR AB(nurs* OR "nurse-led" OR "nurse led" OR "nurse-driven"

OR "nurse-initiated" OR "registered nurse" OR "registered nurses"

OR "nurse practitioner" OR "nurse practitioners"

OR "advanced practice nurse" OR "advanced practice nursing"

OR "clinical nurse specialist" OR "clinical nurse specialists"

OR "nursing staff" OR "nursing personnel" OR "bedside nurse*"

OR "ward nurse*" OR "ICU nurse*" OR "critical care nurse*"

OR "infection control nurse*" OR "infection prevention nurse*"

OR "primary care nurse*" OR "community nurse*"

OR "nurse prescrib*" OR "advanced nurse practitioner*")

OR (MH "Nurses") OR (MH "Nursing Staff, Hospital")

OR (MH "Nurse Practitioners") OR (MH "Advanced Practice Nursing")

OR (MH "Clinical Nurse Specialists") )

AND

( TI("antimicrobial stewardship" OR "antibiotic stewardship"

OR "antimicrobial stewardship program*" OR "antibiotic stewardship program*"

OR "antibiotic management" OR "antimicrobial management"

OR "rational antibiotic use" OR "appropriate antibiotic use"

OR "appropriate antimicrobial use" OR "antibiotic optimi*"

OR "antimicrobial optimi*" OR "antibiotic timeout" OR "antibiotic time-out"

OR "IV-to-oral" OR "IV-to-PO" OR "intravenous to oral" OR "parenteral to oral"

OR "de-escalation" OR "antibiotic de-escalation" OR "antimicrobial de-escalation"

OR "prospective audit and feedback" OR "antimicrobial audit" OR "antibiotic audit"

OR "antibiotic allergy" OR "penicillin allergy"

OR "allergy de-labelling" OR "allergy delabelling"

OR "antibiotic prescribing" OR "antimicrobial prescribing"

OR "formulary restriction" OR "prior authoris*" OR "prior authoriz*"

OR "sepsis bundle" OR "antibiotic review" OR "antimicrobial review"

OR "medication reconciliation")

OR AB("antimicrobial stewardship" OR "antibiotic stewardship"

OR "antimicrobial stewardship program*" OR "antibiotic stewardship program*"

OR "antibiotic management" OR "antimicrobial management"

OR "rational antibiotic use" OR "appropriate antibiotic use"

OR "appropriate antimicrobial use" OR "antibiotic optimi*"

OR "antimicrobial optimi*" OR "antibiotic timeout" OR "antibiotic time-out"

OR "IV-to-oral" OR "IV-to-PO" OR "intravenous to oral" OR "parenteral to oral"

OR "de-escalation" OR "antibiotic de-escalation" OR "antimicrobial de-escalation"

OR "prospective audit and feedback" OR "antimicrobial audit" OR "antibiotic audit"

OR "antibiotic allergy" OR "penicillin allergy"

OR "allergy de-labelling" OR "allergy delabelling"

OR "antibiotic prescribing" OR "antimicrobial prescribing"

OR "formulary restriction" OR "prior authoris*" OR "prior authoriz*"

OR "sepsis bundle" OR "antibiotic review" OR "antimicrobial review"

OR "medication reconciliation")

OR (MH "Antimicrobial Stewardship") OR (MH "Anti-Infective Agents") )

AND PY 2000-2026

Search 2: CFIR Supplement (Block A AND Block B AND Block D)

( TI(nurs* OR "nurse-led" OR "nurse led" OR "nurse-driven"

OR "nurse-initiated" OR "registered nurse" OR "registered nurses"

OR "nurse practitioner" OR "nurse practitioners"

OR "advanced practice nurse" OR "advanced practice nursing"

OR "clinical nurse specialist" OR "clinical nurse specialists"

OR "nursing staff" OR "nursing personnel" OR "bedside nurse*"

OR "ward nurse*" OR "ICU nurse*" OR "critical care nurse*"

OR "infection control nurse*" OR "infection prevention nurse*"

OR "primary care nurse*" OR "community nurse*"

OR "nurse prescrib*" OR "advanced nurse practitioner*")

OR AB(nurs* OR "nurse-led" OR "nurse led" OR "nurse-driven"

OR "nurse-initiated" OR "registered nurse" OR "registered nurses"

OR "nurse practitioner" OR "nurse practitioners"

OR "advanced practice nurse" OR "advanced practice nursing"

OR "clinical nurse specialist" OR "clinical nurse specialists"

OR "nursing staff" OR "nursing personnel" OR "bedside nurse*"

OR "ward nurse*" OR "ICU nurse*" OR "critical care nurse*"

OR "infection control nurse*" OR "infection prevention nurse*"

OR "primary care nurse*" OR "community nurse*"

OR "nurse prescrib*" OR "advanced nurse practitioner*")

OR (MH "Nurses") OR (MH "Nursing Staff, Hospital")

OR (MH "Nurse Practitioners") OR (MH "Advanced Practice Nursing")

OR (MH "Clinical Nurse Specialists") )

AND

( TI("antimicrobial stewardship" OR "antibiotic stewardship"

OR "antimicrobial stewardship program*" OR "antibiotic stewardship program*"

OR "antibiotic management" OR "antimicrobial management"

OR "rational antibiotic use" OR "appropriate antibiotic use"

OR "appropriate antimicrobial use" OR "antibiotic optimi*"

OR "antimicrobial optimi*" OR "antibiotic timeout" OR "antibiotic time-out"

OR "IV-to-oral" OR "IV-to-PO" OR "intravenous to oral" OR "parenteral to oral"

OR "de-escalation" OR "antibiotic de-escalation" OR "antimicrobial de-escalation"

OR "prospective audit and feedback" OR "antimicrobial audit" OR "antibiotic audit"

OR "antibiotic allergy" OR "penicillin allergy"

OR "allergy de-labelling" OR "allergy delabelling"

OR "antibiotic prescribing" OR "antimicrobial prescribing"

OR "formulary restriction" OR "prior authoris*" OR "prior authoriz*"

OR "sepsis bundle" OR "antibiotic review" OR "antimicrobial review"

OR "medication reconciliation")

OR AB("antimicrobial stewardship" OR "antibiotic stewardship"

OR "antimicrobial stewardship program*" OR "antibiotic stewardship program*"

OR "antibiotic management" OR "antimicrobial management"

OR "rational antibiotic use" OR "appropriate antibiotic use"

OR "appropriate antimicrobial use" OR "antibiotic optimi*"

OR "antimicrobial optimi*" OR "antibiotic timeout" OR "antibiotic time-out"

OR "IV-to-oral" OR "IV-to-PO" OR "intravenous to oral" OR "parenteral to oral"

OR "de-escalation" OR "antibiotic de-escalation" OR "antimicrobial de-escalation"

OR "prospective audit and feedback" OR "antimicrobial audit" OR "antibiotic audit"

OR "antibiotic allergy" OR "penicillin allergy"

OR "allergy de-labelling" OR "allergy delabelling"

OR "antibiotic prescribing" OR "antimicrobial prescribing"

OR "formulary restriction" OR "prior authoris*" OR "prior authoriz*"

OR "sepsis bundle" OR "antibiotic review" OR "antimicrobial review"

OR "medication reconciliation")

OR (MH "Antimicrobial Stewardship") OR (MH "Anti-Infective Agents") )

AND

( TI("implementation science" OR "implementation research"

OR "Consolidated Framework for Implementation Research"

OR "CFIR" OR "CFIR 2.0" OR "implementation barriers"

OR "implementation facilitators" OR "implementation determinants"

OR "implementation fidelity" OR "Normalisation Process Theory" OR NPT

OR "knowledge translation" OR "organisational readiness"

OR "organizational readiness")

OR AB("implementation science" OR "implementation research"

OR "Consolidated Framework for Implementation Research"

OR "CFIR" OR "CFIR 2.0" OR "implementation barriers"

OR "implementation facilitators" OR "implementation determinants"

OR "implementation fidelity" OR "Normalisation Process Theory" OR NPT

OR "knowledge translation" OR "organisational readiness"

OR "organizational readiness" OR interview* OR "focus group*"

OR "qualitative stud*" OR "stakeholder perception*")

OR (MH "Implementation Science") OR (MH "Qualitative Research") )

AND PY 2000-2026

**Database 4: Cochrane CENTRAL Register of Controlled Trials**

Primary Search (Block A AND Block B)

(nurs* OR "nurse-led" OR "nurse-driven" OR "nurse-initiated" | | OR "registered nurse" OR "registered nurses" | | OR "nurse practitioner" OR "nurse practitioners" | | OR "advanced practice nurse" OR "advanced practice nursing" | | OR "clinical nurse specialist" OR "clinical nurse specialists" | | OR "nursing staff" OR "nursing personnel" OR "bedside nurse*" | | OR "ward nurse*" OR "ICU nurse*" OR "critical care nurse*" | | OR "infection control nurse*" OR "infection prevention nurse*" | | OR "primary care nurse*" OR "community nurse*" | | OR "nurse prescrib*" OR "advanced nurse practitioner*") | | AND | | ("antimicrobial stewardship" OR "antibiotic stewardship" | | OR "antimicrobial stewardship program*" OR "antibiotic stewardship program*" | | OR "antibiotic management" OR "antimicrobial management" | | OR "rational antibiotic use" OR "appropriate antibiotic use" | | OR "appropriate antimicrobial use" | | OR "antibiotic optimis*" OR "antibiotic optimiz*" | | OR "antimicrobial optimis*" OR "antimicrobial optimiz*" | | OR "antibiotic timeout" OR "antibiotic time-out" | | OR "antimicrobial timeout" OR "antimicrobial time-out" | | OR "IV-to-oral" OR "IV-to-PO" OR "intravenous to oral" OR | "parenteral to oral" | | OR "de-escalation" OR "antibiotic de-escalation" OR | "antimicrobial de-escalation" | | OR "prospective audit and feedback" OR "antimicrobial audit" OR | "antibiotic audit" | | OR "antibiotic allergy" OR "penicillin allergy" | | OR "allergy de-labelling" OR "allergy delabelling" | | OR "penicillin de-labelling" OR "penicillin delabelling" | | OR "antibiotic prescribing" OR "antimicrobial prescribing" | | OR "formulary restriction" OR "prior authoris*" OR "prior | authoriz*" | | OR "sepsis bundle" OR "antibiotic review" OR "antimicrobial | review" | | OR "medication reconciliation") | | [Apply date filter: Year 2000 to current in filter panel] |

Note: Cochrane CENTRAL does not support MeSH explosion via free-text search; controlled vocabulary terms from Block A/B are therefore covered through the free-text wildcard terms above.

**Database 5: Scopus**

Search 1: Primary (Block A AND Block B)

TITLE-ABS-KEY( nurs* OR "nurse-led" OR "nurse led" OR "nurse-driven"

OR "nurse-initiated" OR "registered nurse" OR "registered nurses"

OR "nurse practitioner" OR "nurse practitioners"

OR "advanced practice nurse" OR "advanced practice nursing"

OR "clinical nurse specialist" OR "clinical nurse specialists"

OR "nursing staff" OR "nursing personnel"

OR "bedside nurse*" OR "ward nurse*"

OR "ICU nurse*" OR "critical care nurse*"

OR "infection control nurse*" OR "infection prevention nurse*"

OR "primary care nurse*" OR "community nurse*"

OR "nurse prescrib*" OR "advanced nurse practitioner*" )

AND TITLE-ABS-KEY( "antimicrobial stewardship" OR "antibiotic stewardship"

OR "antimicrobial stewardship program*" OR "antimicrobial stewardship programme"

OR "antibiotic stewardship program*" OR "antibiotic stewardship programme"

OR "antibiotic management" OR "antimicrobial management"

OR "rational antibiotic use" OR "appropriate antibiotic use"

OR "appropriate antimicrobial use"

OR "antibiotic optimis*" OR "antibiotic optimiz*" OR "antimicrobial optimis*" OR "antimicrobial optimiz*"

OR "antibiotic timeout" OR "antibiotic time-out"

OR "antimicrobial timeout" OR "IV-to-oral" OR "IV-to-PO"

OR "intravenous to oral" OR "parenteral to oral"

OR "de-escalation" OR "antibiotic de-escalation" OR "antimicrobial de-escalation"

OR "prospective audit and feedback" OR "antimicrobial audit" OR "antibiotic audit"

OR "antibiotic allergy" OR "penicillin allergy"

OR "allergy de-labelling" OR "allergy delabelling"

OR "antibiotic prescribing" OR "antimicrobial prescribing"

OR "formulary restriction" OR "prior authoris*" OR "prior authoriz*"

OR "sepsis bundle" OR "antibiotic review" OR "antimicrobial review"

OR "medication reconciliation" )

AND PUBYEAR > 1999

AND DOCTYPE ( ar OR re OR cp )

Search 2: CFIR Supplement (Block A AND Block B AND Block D)

AND TITLE-ABS-KEY( "implementation science" OR "implementation research"

OR "Consolidated Framework for Implementation Research"

OR CFIR OR "CFIR 2.0"

OR "implementation barriers" OR "implementation facilitators"

OR "implementation determinants" OR "implementation fidelity"

OR "Normalisation Process Theory" OR NPT

OR "knowledge translation" OR "organisational readiness"

OR "organizational readiness" OR interview*

OR "focus group" OR "qualitative stud" OR "stakeholder perception" )

Search 3: GCC/MENA Supplement (Block A AND Block B AND Block F)

AND TITLE-ABS-KEY( "Gulf Cooperation Council" OR GCC

OR "Middle East and North Africa" OR MENA OR "Eastern Mediterranean"

OR "Saudi Arabia" OR "Kingdom of Saudi Arabia" OR KSA

OR "United Arab Emirates" OR UAE OR "Abu Dhabi" OR Dubai

OR Qatar OR Kuwait OR Bahrain OR Oman

OR Jordan OR Lebanon OR Egypt OR Syria

OR Iraq OR Iran OR Morocco OR Tunisia

OR Algeria OR Libya OR Sudan OR Yemen

OR "Arab world" OR "Arab countries" OR "WHO EMRO" )

**Database 6: Web of Science Core Collection**

Search 1: Primary (Block A AND Block B)

TS=(nurs* OR "nurse-led" OR "nurse led" OR "nurse-driven"

OR "nurse-initiated" OR "registered nurse" OR "registered nurses"

OR "nurse practitioner" OR "nurse practitioners"

OR "advanced practice nurse" OR "advanced practice nursing"

OR "clinical nurse specialist" OR "clinical nurse specialists"

OR "nursing staff" OR "nursing personnel" OR "bedside nurse*"

OR "ward nurse*" OR "ICU nurse*" OR "critical care nurse*"

OR "infection control nurse*" OR "infection prevention nurse*"

OR "primary care nurse*" OR "community nurse*"

OR "nurse prescrib*" OR "advanced nurse practitioner*")

AND

TS=("antimicrobial stewardship" OR "antibiotic stewardship"

OR "antimicrobial stewardship program*" OR "antibiotic stewardship program*"

OR "antibiotic management" OR "antimicrobial management"

OR "rational antibiotic use" OR "appropriate antibiotic use"

OR "appropriate antimicrobial use" OR "antibiotic optimi*"

OR "antimicrobial optimi*" OR "antibiotic timeout" OR "antibiotic time-out"

OR "antimicrobial timeout" OR "IV-to-oral" OR "IV-to-PO"

OR "intravenous to oral" OR "parenteral to oral"

OR "de-escalation" OR "antibiotic de-escalation" OR "antimicrobial de-escalation"

OR "prospective audit and feedback" OR "antimicrobial audit" OR "antibiotic audit"

OR "antibiotic allergy" OR "penicillin allergy"

OR "allergy de-labelling" OR "allergy delabelling"

OR "antibiotic prescribing" OR "antimicrobial prescribing"

OR "formulary restriction" OR "prior authoris*" OR "prior authoriz*"

OR "sepsis bundle" OR "antibiotic review" OR "antimicrobial review"

OR "medication reconciliation")

AND PY=(2000-2026)

Search 2: CFIR Supplement (Block A AND Block B AND Block D)

AND

TS=("implementation science" OR "implementation research"

OR "Consolidated Framework for Implementation Research"

OR CFIR OR "CFIR 2.0" OR "implementation barriers"

OR "implementation facilitators" OR "implementation determinants"

OR "implementation fidelity" OR "Normalisation Process Theory" OR NPT

OR "knowledge translation" OR "organisational readiness"

OR "organizational readiness" OR interview* OR "focus group*"

OR "qualitative stud*" OR "stakeholder perception*")

Search 3: GCC/MENA Supplement (Block A AND Block B AND Block F)

AND

TS=("Gulf Cooperation Council" OR GCC

OR "Middle East and North Africa" OR MENA OR "Eastern Mediterranean"

OR "Saudi Arabia" OR "Kingdom of Saudi Arabia" OR KSA

OR "United Arab Emirates" OR UAE OR "Abu Dhabi" OR Dubai

OR Qatar OR Kuwait OR Bahrain OR Oman

OR Jordan OR Lebanon OR Egypt OR Syria

OR Iraq OR Iran OR Morocco OR Tunisia

OR Algeria OR Libya OR Sudan OR Yemen

OR "Arab world" OR "Arab countries" OR "WHO EMRO")

AND PY=(2000-2026)

**Database 7: WHO Global Index Medicus**

Search 1: Primary

(nurs* OR "nurse-led" OR "nurse led" OR "registered nurse"

OR "nurse practitioner" OR "advanced practice nurse"

OR "clinical nurse specialist" OR "nursing staff"

OR "bedside nurse*" OR "ward nurse*" OR "ICU nurse*"

OR "infection control nurse*" OR "community nurse*"

OR "nurse prescrib*" OR "advanced nurse practitioner*")

AND

("antimicrobial stewardship" OR "antibiotic stewardship"

OR "antibiotic management" OR "antimicrobial management"

OR "rational antibiotic use" OR "appropriate antibiotic use"

OR "antibiotic optimis*" OR "antibiotic optimiz*" OR "antimicrobial optimis*" OR "antimicrobial optimiz*"

OR "antibiotic prescribing" OR "antimicrobial prescribing"

OR "IV-to-oral" OR "de-escalation" OR "antibiotic timeout"

OR "antimicrobial audit" OR "prospective audit and feedback"

OR "penicillin allergy" OR "antibiotic allergy"

OR "formulary restriction" OR "antibiotic review" OR "antimicrobial review")

[Date filter: 2000 to current year]

Search 2: GCC/MENA Supplement

AND ("Gulf Cooperation Council" OR GCC OR "Middle East" OR MENA

OR "Eastern Mediterranean" OR "Saudi Arabia" OR UAE OR "Abu Dhabi"

OR Qatar OR Kuwait OR Bahrain OR Oman OR Jordan OR Lebanon

OR Egypt OR Syria OR Iraq OR Iran OR Morocco OR Tunisia

OR Algeria OR Libya OR Sudan OR Yemen OR "Arab world")

**Database 8: LILACS**

Primary Search

(nurs* OR "nurse-led" OR "registered nurse" OR "nurse practitioner"

OR "advanced practice nurse" OR "clinical nurse specialist"

OR "nursing staff" OR "infection control nurse*" OR "nurse prescrib*")

AND

("antimicrobial stewardship" OR "antibiotic stewardship"

OR "antibiotic management" OR "antimicrobial management"

OR "rational antibiotic use" OR "appropriate antibiotic use"

OR "antibiotic optimis*" OR "antibiotic optimiz*" OR "antimicrobial optimis*" OR "antimicrobial optimiz*"

OR "antibiotic prescribing" OR "antimicrobial prescribing"

OR "de-escalation" OR "antibiotic timeout"

OR "prospective audit and feedback" OR "antimicrobial audit"

OR "antibiotic review" OR "antimicrobial review")

[Date filter: 2000 to current year]

**Databases 9–11: Trial Registries and Grey Literature**

Run all four keyword searches below using the Advanced Search interface. These databases do not support Boolean strings — use the specific fields indicated.

S1 "antimicrobial stewardship" "nurse-led"

S2 "antibiotic stewardship" nurses hospital

S3 "nurse-led" "antimicrobial stewardship" barriers OR facilitators

S4 "nursing staff" "antimicrobial stewardship" OR "antibiotic stewardship" outcomes
