# Supplementary material for: Nurse-Led and Nurse-Involved Antimicrobial Stewardship in Hospitals and Primary Care: Protocol for a Mixed Methods Systematic Review and Meta-Analysis With Gulf Cooperation Council and Middle East and North Africa Subgroup Analysis
Source: JMIR Res Protoc. 2026 Oct 8;15:e96342. doi: 10.2196/96342 (PMC13649170; doi:10.2196/96342)
Supplement: Supplementary file 4 [file resprot-v15-e96342-s003.docx]

**Multimedia Appendix 3 – Quantitative Data Extraction Form & Quality Appraisal: Stream 1**

INSTRUCTIONS FOR REVIEWERS:

1. Use for: All studies included in the quantitative meta-analysis and narrative synthesis (Stream 1).
2. Complete all fields for every included study.
3. For outcomes data: enter the exact numbers reported — do not calculate or transform values here.
4. If a field cannot be extracted (data not reported), enter 'NR' (Not Reported).
5. For verbatim text fields, copy the exact wording from the paper — do not paraphrase.
6. GCC/MENA coding is based solely on the country where the study was conducted, not author affiliation.
7. Each extracted field will be independently reviewed and compared between the two reviewers during the kappa calibration meeting.

**Section A: Study Identification**

| A1. Study/Covidence ID  Unique ID |  |
| --- | --- |
| A2. Author(s)  Last name + initials of all authors |  |
| A3. Year of publication  Four-digit year. For online-first publications, use year of online publication |  |
| A4. Country/Countries  Country where the intervention was delivered — not author affiliation country |  |
| A5. Language  Language of publication (English / French / Spanish / Arabic / Other: specify) |  |
| A6. Journal name  Full journal name |  |
| A7. Study design  Select one: RCT / Cluster RCT / Stepped-wedge RCT / Quasi-experimental / ITS / CBA / UBA |  |
| A8. Setting type  Select one or more: Hospital inpatient / ICU / Emergency department / Primary care / Community / Other: specify |  |
| A9. Hospital type  Select: Teaching/tertiary / District general / Community / Primary care / Mixed / NR |  |
| A10. Study duration  Total duration of the study including follow-up period (e.g., 12 months pre + 6 months post) |  |

**Section B: Population Details**

| B1. Patient population  Brief description: age range, diagnosis, clinical condition (e.g., adult inpatients receiving antimicrobials) |  |
| --- | --- |
| B2. Total patient sample size  Number of patients in the study (pre + post, or intervention + control) |  |
| B3. Nurse population  Description of nursing staff who delivered the intervention (e.g., RNs, CNS, ward nurses — use terminology from paper) |  |
| B4. Number of nurses  Number of nurses involved, if reported. If not reported: 'NR' |  |
| B5. Nurse training  Was specific AMS training provided to nurses? Yes / No / Partially described / NR |  |

**Section C: Nurse-Led AMS Intervention**

| C1. Nurse role description  Verbatim from the paper — copy the exact sentence(s) describing the nurse's AMS role |  |
| --- | --- |
| C2. Intervention type  Select all that apply: Antibiotic timeout / IV-to-oral switch / Blood culture protocol / Patient education / Allergy de-labelling / Prescribing audit and feedback / De-escalation support / Medication reconciliation / Sepsis bundle / Other: specify |  |
| C3. Nurse autonomy level  Select: Fully nurse-led (nurse is primary decision-maker) / Semi-autonomous (nurse initiates; prescriber decides) / Nurse-involved within MDT programme (nurse performs defined task within physician-led ASP) / Unclear |  |
| C4. Intervention components  List all distinct components (e.g., 1. 48-hr timeout prompt; 2. IV-to-oral assessment checklist; 3. monthly feedback to ward) |  |
| C5. Frequency / duration  How often the intervention was delivered and over what time period |  |
| C6. Implementation tools used  e.g., electronic alert, paper checklist, bedside prompt card, protocol document — specify |  |
| C7. Multidisciplinary involvement  Were pharmacists, physicians, or microbiologists also involved? Yes / No / Partially — describe |  |

**Section D: Comparator/Control**

| D1. Comparator type  Select: Pre-intervention (same ward/hospital) / Concurrent control ward / Concurrent control hospital / No comparator (UBA) / Historical comparator / Other: specify |  |
| --- | --- |
| D2. Comparator description  Brief description of what was the standard of care in the control condition |  |

**Section E: Primary Quantitative Outcomes (Stream 1)**

Extract all outcomes reported — do not select. For outcomes not reported in this study, enter 'NR'. For outcomes reported but not quantifiable, enter 'Qualitative only'.

| E1. Antibiotic consumption  Metric used (DOT per 1,000 patient-days / DDD per 1,000 patient-days / total units / other). Pre-intervention value: [x] / Post-intervention value: [x] / p-value: / 95% CI: / Effect size (if reported): |  |
| --- | --- |
| E2. Prescribing appropriateness  Metric (% appropriate / error rate / guideline adherence score / AWaRe classification). Pre: [x] / Post: [x] / p-value: / 95% CI: |  |
| E3. Clinical patient outcomes  Report separately: (a) All-cause in-hospital mortality [%]: Pre / Post / p / CI; (b) Infection-attributed mortality [%]: Pre / Post / p / CI; (c) Hospital LOS [days, mean or median]: Pre / Post; (d) ICU LOS [days]: Pre / Post |  |
| E4. Time to first antibiotic dose  Hours from assessment/sepsis recognition to first dose. Pre: / Post: / p: / CI: |  |
| E5. Blood culture collection rate  % of eligible patients with blood culture before first antibiotic. Pre: / Post: / p: / CI: |  |

**Section F: Secondary Quantitative Outcomes**

| F1. CDI rate  Clostridium/Clostridioides difficile infection rate. Values + statistics if reported, or NR |  |
| --- | --- |
| F2. HAI rates  CAUTI / VAP / CLABSI rates — specify which, report values |  |
| F3. MDRO isolation rates  MRSA / CRE / ESBL — specify organism, report values |  |
| F4. 30-day readmission rates  All-cause or infection-related — specify. Values + statistics |  |
| F5. Antibiotic-related cost savings  Direct drug costs — specify currency and reference year. Values + statistics |  |
| F6. Nurse AMS compliance rate  % of eligible clinical encounters where nurse performed mandated AMS task. Values + statistics |  |

**Section G: Quality Appraisal — Risk of Bias**

Tool assignment: Select the tool that matches this study's design. Complete ONLY the relevant sub-section. Leave others blank.

| G0. Study design confirmed  Re-confirm: RCT / Cluster RCT / Quasi-experimental / CBA / UBA / ITS |  |
| --- | --- |
| G0a. Appraisal tool applied  Select: Cochrane RoB 2 (for RCTs) / ROBINS-I (for quasi-experimental, CBA, UBA) / EPOC ITS criteria (for ITS) |  |

**G1: Cochrane Risk of Bias 2 (RoB 2) – Complete ONLY for RCTs and cluster-RCTs**

Each domain is rated: Low risk / Some concerns / High risk. Overall judgement follows.

| G1.1 Domain 1 — Randomisation process  Was the allocation sequence random? Was allocation concealed? Any baseline imbalance? Rating: Low / Some concerns / High |  |
| --- | --- |
| G1.2 Domain 2 — Deviations from intended interventions  Were participants and personnel blinded? Were there unintended deviations from the protocol? Rating: Low / Some concerns / High |  |
| G1.3 Domain 3 — Missing outcome data  Were outcome data available for all or nearly all participants? Was missingness related to true outcome? Rating: Low / Some concerns / High |  |
| G1.4 Domain 4 — Measurement of the outcome  Was the outcome measurement method appropriate? Were assessors blinded to intervention assignment? Rating: Low / Some concerns / High |  |
| G1.5 Domain 5 — Selection of reported results  Are the reported results consistent with the pre-registered analysis plan? Rating: Low / Some concerns / High |  |
| G1.6 Overall RoB 2 judgement  Determined by the highest-rated domain: Low / Some concerns / High |  |
| G1.7 Notes  Describe specific concerns per domain. Note impact on GRADE certainty rating. |  |

**G2: ROBINS-I – Complete ONLY for quasi-experimental, CBA, and UBA studies**

Each domain: Low / Moderate / Serious / Critical / No information (NI). Overall determined by the highest-rated domain.

| G2.1 Domain 1 — Bias due to confounding  Were important confounders measured and adjusted for? Was the pre-intervention period comparable? Rating: Low / Moderate / Serious / Critical / NI |  |
| --- | --- |
| G2.2 Domain 2 — Bias in selection of participants  Were all eligible participants included? Was selection related to the outcome? Rating: Low / Moderate / Serious / Critical / NI |  |
| G2.3 Domain 3 — Bias in classification of interventions  Was the nurse AMS intervention clearly defined and distinguished from comparator? Was misclassification possible? Rating: Low / Moderate / Serious / Critical / NI |  |
| G2.4 Domain 4 — Bias due to deviations from intended interventions  Did participants or staff deviate from the intended protocol? Was co-intervention applied differentially? Rating: Low / Moderate / Serious / Critical / NI |  |
| G2.5 Domain 5 — Bias due to missing data  Were outcome data reasonably complete? Was missingness related to the intervention or outcome? Rating: Low / Moderate / Serious / Critical / NI |  |
| G2.6 Domain 6 — Bias in measurement of outcomes  Was the outcome measured using valid, reliable instruments? Could assessors have been influenced by knowledge of the intervention? Rating: Low / Moderate / Serious / Critical / NI |  |
| G2.7 Domain 7 — Bias in selection of reported results  Are all pre-specified outcomes reported? Evidence of outcome switching or selective reporting? Rating: Low / Moderate / Serious / Critical / NI |  |
| G2.8 Overall ROBINS-I judgement  Low / Moderate / Serious / Critical |  |
| G2.9 Notes  Describe specific concerns per domain. Note impact on GRADE certainty rating. |  |

**G3: EPOC ITS Criteria — Complete ONLY for Interrupted Time Series studies**

Each item: Yes / No / Unclear. Overall quality: Strong / Moderate / Weak.

| G3.1 Was the intervention independent of other changes?  Could the observed changes be due to a concurrent event rather than the AMS intervention? Yes / No / Unclear |  |
| --- | --- |
| G3.2 Was the shape of the intervention effect pre-specified?  Was the expected direction and timing of the effect described before data collection? Yes / No / Unclear |  |
| G3.3 Were intervention unlikely to affect data collection?  Could the intervention have altered how the outcomes were measured or recorded? Yes / No / Unclear |  |
| G3.4 Were data collected regularly over time?  Is there evidence that data were recorded at consistent, regular intervals? Yes / No / Unclear |  |
| G3.5 Minimum 3 data points before and 3 after intervention?  Were at least 3 clearly defined time-points available on each side of the intervention? Yes / No / Unclear |  |
| G3.6 Was a contemporaneous control group available?  Was there a parallel control unit or time series not exposed to the intervention? Yes / No / Unclear |  |
| G3.7 Was the outcome assessed in a standardised way?  Were the same measurement instruments and data sources used consistently throughout? Yes / No / Unclear |  |
| G3.8 Overall ITS quality  Strong (6–7 Yes) / Moderate (4–5 Yes) / Weak (fewer than 4 Yes) |  |
| G3.9 Notes  Describe specific concerns. Note whether the absence of a control group will affect narrative synthesis conclusions. |  |

**Section H: GCC/MENA Coding and Miscellaneous**

| H1. GCC/MENA relevance  Select: YES (study conducted exclusively in GCC/MENA country) / PARTIAL (multi-country study with at least one GCC/MENA site) / NO (conducted entirely outside GCC/MENA). Coding is based on country of study conduct only, not author affiliation. |  |
| --- | --- |
| H2. GCC/MENA country (if Yes/Partial)  Specify: Saudi Arabia / UAE / Qatar / Kuwait / Bahrain / Oman / Jordan / Lebanon / Egypt / Iran / Iraq / Syria / Palestine / Yemen / Other MENA country |  |
| H3. Possible studies for inclusion  Does this study's reference list contain other potentially eligible studies not yet in our database? Yes (list): / No |  |
| H4. Extractor ID  Initials of reviewer completing this extraction |  |
| H5. Extraction date  DD/MM/YYYY |  |
| H6. Notes / queries for team meeting  Any uncertainty, ambiguity, or question to raise at the calibration or extraction meeting |  |
