# Supplementary material for: Nurse-Led and Nurse-Involved Antimicrobial Stewardship in Hospitals and Primary Care: Protocol for a Mixed Methods Systematic Review and Meta-Analysis With Gulf Cooperation Council and Middle East and North Africa Subgroup Analysis
Source: JMIR Res Protoc. 2026 Oct 8;15:e96342. doi: 10.2196/96342 (PMC13649170; doi:10.2196/96342)
Supplement: Supplementary file 5 [file resprot-v15-e96342-s004.docx]

**Multimedia Appendix 3 – Qualitative Data Extraction Form & Quality Appraisal: Stream 2**

INSTRUCTIONS FOR REVIEWERS:

1. Use for: All qualitative, mixed-methods, and survey studies included in the implementation synthesis (Stream 2).
2. Read the full paper before beginning extraction — do not extract from the abstract alone.
3. For each CFIR domain, record: (a) direct quotes from the paper; (b) your thematic summary of those quotes; (c) barrier or facilitator classification.
4. A single quote may be relevant to more than one CFIR domain — record it in all applicable domains.
5. If a CFIR domain is not addressed by the study, enter 'Not addressed in this study' — do not leave blank.
6. The boundary between CFIR domains (especially Inner Setting vs Individuals) is sometimes ambiguous — record in the most appropriate domain and note the ambiguity.
7. For surveys: treat reported percentages and frequencies as 'findings' in the relevant domain, not as quotes.

**Section A: Study Identification**

| A1. Study/Covidence ID  Unique ID |  |
| --- | --- |
| A2. Author(s)  Last name + initials of all authors |  |
| A3. Year  Four-digit year |  |
| A4. Country/Countries  Country where the study was conducted — not author affiliation |  |
| A5. Language  Language of publication |  |
| A6. Study method  Select: Semi-structured interviews / Focus groups / Survey / Ethnography / Action research / Grounded theory / Thematic analysis / Mixed-methods / Other: specify |  |
| A7. Setting type  Hospital inpatient ward / ICU / Primary care / Community / Mixed / Multiple settings / Unspecified |  |
| A8. Health system context  Brief description: public/private, country income level, hospital accreditation status if reported |  |

**Section B: Population Details**

| B1. Participant roles  Tick all that apply: Registered Nurses / Clinical Nurse Specialists / Nurse Practitioners / Ward Nurses / ICU Nurses / Infection Control Nurses / Physicians / Pharmacists / Microbiologists / Managers/Administrators / Patients / Mixed healthcare staff / Other: specify |  |
| --- | --- |
| B2. Total sample size  Total number of participants |  |
| B3. Nurse sample size  Number of nurses specifically — if mixed sample |  |
| B4. Sampling method  Purposive / Convenience / Snowball / Random / NR |  |
| B5. Data saturation reached  Yes / No / Not reported / Not applicable (survey) |  |

**Section C: CFIR 2.0 Domain Coding**

For each domain: record direct quotes → write thematic summary → classify as BARRIER, FACILITATOR, or BOTH.

KEY: B = Barrier to nurse-led AMS implementation | F = Facilitator | B/F = Both present in same study

Quote format: 'exact quote from paper' (participant role, page/paragraph number if available)

Thematic summary: your own words — what does this quote tell us about why nurse-led AMS works or fails in this context?

| **CFIR Domain 1 — Innovation Characteristics**  **Attributes of the AMS intervention itself that influence whether it is adopted and delivered as intended** | |
| --- | --- |
| C1.1 Direct quotes from study  Copy exact quotes relevant to this domain. Include participant role where reported. Format: "quote here" (Nurse, p.X) |  |
| C1.2 Thematic summary  Your own words: what does the evidence from this study tell us about this CFIR domain in relation to nurse-led AMS? |  |
| C1.3 Classification  Select: BARRIER only / FACILITATOR only / BOTH BARRIER AND FACILITATOR present / Not addressed in this study |  |
| C1.4 Specific construct(s) within domain  Identify which CFIR 2.0 constructs are relevant. e.g., Relative Advantage ('timeout is seen as an improvement on current practice'), Complexity ('nurses find the IV-to-oral criteria too complex'), Adaptability ('the protocol was modified for the ICU context') |  |
| **CFIR Domain 2 — Outer Setting**  **External contextual factors: national AMR policies, accreditation, regulatory requirements, external peer pressure, broader epidemiology** | |
| C2.1 Direct quotes from study  Copy exact quotes relevant to this domain. Include participant role where reported. Format: "quote here" (Nurse, p.X) |  |
| C2.2 Thematic summary  Your own words: what does the evidence from this study tell us about this CFIR domain in relation to nurse-led AMS? |  |
| C2.3 Classification  Select: BARRIER only / FACILITATOR only / BOTH BARRIER AND FACILITATOR present / Not addressed in this study |  |
| C2.4 Specific construct(s) within domain  Identify which CFIR 2.0 constructs are relevant. e.g., External Policy ('national AMR strategy mandated stewardship'), Accreditation ('JCI accreditation required a nurse AMS role'), Patient Population ('high MDRO prevalence drove urgency') |  |

| **CFIR Domain 3 — Inner Setting**  **Organisational characteristics: hospital culture, leadership support, resources, communication structures, professional hierarchies** | |
| --- | --- |
| C3.1 Direct quotes from study  Copy exact quotes relevant to this domain. Include participant role where reported. Format: "quote here" (Nurse, p.X) |  |
| C3.2 Thematic summary  Your own words: what does the evidence from this study tell us about this CFIR domain in relation to nurse-led AMS? |  |
| C3.3 Classification  Select: BARRIER only / FACILITATOR only / BOTH BARRIER AND FACILITATOR present / Not addressed in this study |  |
| C3.4 Specific construct(s) within domain  Identify which CFIR 2.0 constructs are relevant. e.g., Leadership Engagement ('ward manager actively supported the timeout'), Culture ('hierarchical culture meant nurses were reluctant to question prescribers'), Structural Characteristics ('no dedicated nurse AMS time'), Communication ('no mechanism to feed back blood culture results to nurses') |  |

| **CFIR Domain 4 — Individuals**  **Characteristics of those delivering and receiving the intervention: knowledge, beliefs, self-efficacy, role identity, motivation** | |
| --- | --- |
| C4.1 Direct quotes from study  Copy exact quotes relevant to this domain. Include participant role where reported. Format: "quote here" (Nurse, p.X) |  |
| C4.2 Thematic summary  Your own words: what does the evidence from this study tell us about this CFIR domain in relation to nurse-led AMS? |  |
| C4.3 Classification  Select: BARRIER only / FACILITATOR only / BOTH BARRIER AND FACILITATOR present / Not addressed in this study |  |
| C4.4 Specific construct(s) within domain  Identify which CFIR 2.0 constructs are relevant. e.g., Knowledge ('nurses lacked pharmacological knowledge to challenge prescribers'), Self-Efficacy ('nurses felt confident checking allergy histories'), Role Identity ('I see AMS as part of my job'), Motivation ('nurses motivated by better patient outcomes') |  |

| **CFIR Domain 5 — Implementation Process**  **Activities undertaken to introduce and sustain the intervention: planning, champions, training, monitoring, evaluation, sustainability** | |
| --- | --- |
| C5.1 Direct quotes from study  Copy exact quotes relevant to this domain. Include participant role where reported. Format: "quote here" (Nurse, p.X) |  |
| C5.2 Thematic summary  Your own words: what does the evidence from this study tell us about this CFIR domain in relation to nurse-led AMS? |  |
| C5.3 Classification  Select: BARRIER only / FACILITATOR only / BOTH BARRIER AND FACILITATOR present / Not addressed in this study |  |
| C5.4 Specific construct(s) within domain  Identify which CFIR 2.0 constructs are relevant. e.g., Engaging ('a nurse champion was identified on each ward'), Planning ('no formal implementation plan was developed'), Executing ('monthly audit and feedback meetings maintained engagement'), Reflecting and Evaluating ('programme was still running at 12 months') |  |

**Section D: Quality Appraisal**

Tool assignment: Select the tool that matches this study's design. Complete ONLY the relevant sub-section. Leave others blank.

| D0. Study design confirmed  Re-confirm: Qualitative / Mixed-methods / Cross-sectional survey |  |
| --- | --- |
| D0a. Appraisal tool applied  Select: JBI Qualitative Research Checklist (for qualitative studies) / MMAT 2018 (for mixed-methods) / JBI Critical Appraisal Checklist for Analytical Cross-Sectional Studies (for surveys) |  |

**D1: JBI Critical Appraisal Checklist for Qualitative Research – Complete ONLY for qualitative studies**

Each criterion: Yes (Y) / No (N) / Unclear (U). Quality does not exclude studies but informs confidence in findings.

| D1. Is there congruity between the stated philosophical perspective and the research methodology? Y/N/U/NA |  |
| --- | --- |
| D2. Is there congruity between the research methodology and the research question? Y/N/U/NA |  |
| D3. Is there congruity between the research methodology and the methods used to collect data? Y/N/U/NA |  |
| D4. Is there congruity between the research methodology and the representation and analysis of data? Y/N/U/NA |  |
| D5. Is there congruity between the research methodology and the interpretation of results? Y/N/U/NA |  |
| D6. Is there a statement locating the researcher culturally or theoretically? Y/N/U/NA |  |
| D7. Is the influence of the researcher on the research, and vice versa, addressed? Y/N/U/NA |  |
| D8. Are participants, and their voices, adequately represented? Y/N/U/NA |  |
| D9. Is the research ethical according to current criteria or, for recent studies, has ethical approval been obtained? Y/N/U/NA |  |
| D10. Do the conclusions drawn in the research report flow from the analysis or interpretation of the data? Y/N/U/NA |  |
| D11. Overall appraisal  Include / Exclude / Seek further information. Note: quality does not exclude studies but informs confidence ratings |  |

**D2: Mixed Methods Appraisal Tool (MMAT 2018) – Complete ONLY for mixed-methods studies**

Each item: Yes (Y) / No (N) / Cannot tell (?). Complete screening questions first; if both No, the study cannot be appraised further

| D2.S1 Screening question 1  Are there clear research questions? Y / N / ? |  |
| --- | --- |
| D2.S2 Screening question 2  Do the collected data allow to address the research questions? Y / N / ? |  |
| NOTE: If both screening questions are No, halt appraisal and record 'Not appraised'. |  |
| D2.1, Is there an adequate rationale for using a mixed-methods design to address the research question? Y / N / ? |  |
| D2.2 Are the different components of the study effectively integrated to answer the research question? Y / N / ? |  |
| D2.3 Are the outputs of the integration of qualitative and quantitative components adequately interpreted? Y / N / ? |  |
| D2.4 Are divergences and inconsistencies between quantitative and qualitative results adequately addressed? Y / N / ? |  |
| D2.5 Do the different components of the study adhere to the quality criteria of each tradition of the methods involved? Y / N / ? |  |
| D2.6 Overall MMAT quality  Count of Yes items (0–5): ___ / 5. Quality level: High (4–5 Yes) / Medium (2–3 Yes) / Low (0–1 Yes) |  |
| D2.7 Notes  Specific concerns about integration adequacy or divergence management: |  |

**D3: JBI Critical Appraisal Checklist for Analytical Cross-Sectional Studies — Complete ONLY for survey/cross-sectional studies**

Each item: Yes (Y) / No (N) / Unclear (U) / Not Applicable (NA).

| D3.1 Were the criteria for inclusion in the sample clearly defined? Y/N/U/NA |  |
| --- | --- |
| D3.2 Were the study subjects and the setting described in detail? Y/N/U/NA |  |
| D3.3 Was the exposure (nurse AMS role or participation) measured in a valid and reliable way? Y/N/U/NA |  |
| D3.4 Were objective, standard criteria used for measurement of the condition (e.g., barriers, facilitators, perceptions)? Y/N/U/NA |  |
| D3.5 Were confounding factors identified and stated? Y/N/U/NA |  |
| D3.6 Were strategies to deal with confounding factors stated? Y/N/U/NA |  |
| D3.7 Were the outcomes (e.g., survey responses, Likert ratings, perception scores) measured in a valid and reliable way? Y/N/U/NA |  |
| D3.8 Was appropriate statistical analysis used? Y/N/U/NA |  |
| D3.9 Overall appraisal  Count Yes items: ___ / 8. Include / Seek further information / Exclude. Notes: |  |

**Section E: GCC/MENA Coding and Notes**

| E1. GCC/MENA relevance  YES / PARTIAL / NO — based on country of study conduct only |  |
| --- | --- |
| E2. GCC/MENA country (if Yes/Partial)  Specify country |  |
| E3. Programme sustainability  Was the AMS programme still operational at 6 months post-implementation? Yes / No / NR. At 12 months? Yes / No / NR |  |
| E4. Programme fidelity  Was the intervention delivered as designed? Fully / Partially / No — describe deviations |  |
| E5. Extractor ID  Initials |  |
| E6. Extraction date  DD/MM/YYYY |  |
| E7. Notes / queries  Ambiguities, cross-domain coding uncertainties, questions for the team meeting |  |
